# Supplementary material for: Chemically tailored block copolymers for highly reliable sub-10-nm patterns by directed self-assembly
Source: Nat Commun. 2024 Jul 6;15:5671. doi: 10.1038/s41467-024-49839-0 (PMC11227500; doi:10.1038/s41467-024-49839-0)
Supplement: Supplementary file 1 — Supplementary Information [file 41467_2024_49839_MOESM1_ESM.pdf]

Supplementary Information

Chemically tailored block copolymers for highly reliable sub-10-nm patterns by directed self-assembly

Shinsuke Maekawa[1], Takehiro Seshimo[2], Takahiro Dazai[2], Kazufumi Sato[2], Kan Hatakeyama-Sato[1], Yuta Nabae[1], Teruaki Hayakawa[*1]

[1]Department of Materials Science and Engineering, School of Materials and Chemical Technology, Tokyo Institute of Technology, Tokyo 152-8552, Japan

[2]Research & Development Department, Tokyo Ohka Kogyo Co., Ltd., Kanagawa 253-0114, Japan

Supplementary Methods

Synthesis of PGMA by Living Anionic Polymerization

All polymerization procedures were conducted after purging with argon. LiCl (42 mg, 0.991 mmol) was added to a 200 mL Schlenk flask and dried under reduced pressure while heated with a heat gun. After cooling to room temperature, THF (60 mL) was added to the flask and then cooled to −78 °C using a cooling bath. A solution of *sec*-BuLi in cyclohexane and *n*-hexane was added until the color changed to yellow, and the mixture was stirred for 10 min. The flask was removed from the cooling bath and allowed to warm to room temperature until the solution became colorless, after which it was re-cooled to −78 °C, and *sec*-BuLi in cyclohexane and *n*-hexane (0.164 mL, 0.200 mmol) was added to initiate the reaction. DPE (0.175 mL, 1.00 mmol) was added and the deep red solution was stirred for 30 min. GMA (2.78 mL, 21.1 mmol) was then added and the solution was stirred for 40 min. The solution became colorless. Degassed methanol (10 mL) was added to the flask to prepare proton-terminated PGMA. The polymer was precipitated in MeOH, filtered, and dried under reduced pressure at 40 °C to obtain a white powder (2.91 g, 97% yield). ^{1}H NMR (400 MHz, $CDCl_3$, δ, ppm): 0.51–1.19 (α-CH_3, PGMA), 1.78–2.16 (-CH_2-C(CH_3)-, PGMA), 2.63 (-CH_2-CH(CH_2)-O-, PGMA), 2.84 (-CH_2-CH(CH_2)-O-, PGMA), 3.23 (-CH_2-CH(CH_2)-O-, PGMA), 3.72–3.91 (-CH_2-CH(CH_2)-O-, PGMA), 4.22–4.40 (-CH_2-CH(CH_2)-O-, PGMA), 7.10–7.24 (aromatic, DPE). ^{13}C NMR (100 MHz, $CDCl_3$, δ, ppm): 16.7, 18.8, 43.9, 44.4, 44.8, 45.1, 45.7, 47.9, 48.1, 48.3, 48.4, 48.5, 49.3, 49.5, 49.7, 49.9, 53.9, 65.7, 176.2, 177.0, 177.3. IR (KBr, v, cm^{-1}): 3438, 3065, 3002, 2948, 2887, 1731, 1636, 1485, 1449, 1389, 1366, 1341, 1257, 1172, 1149, 1075, 993, 966, 946, 907, 848, 760, 703, 596, 543.

Synthesis of PGMA-*r*-PMMA9-22 (PGM9-22) by Living Anionic Polymerization

All polymerization procedures were conducted after purging with argon. LiCl (80 mg, 1.89 mmol) was added to a 200 mL Schlenk flask and dried under reduced pressure while heated with a heat gun. After cooling to room temperature, THF (40 mL) was added to the flask and then cooled to −78 °C using a cooling bath. A solution of *sec*-BuLi in cyclohexane and *n*-hexane was added until the color changed to yellow, and the mixture was stirred for 10 min. The flask was removed from the cooling bath and allowed to warm to room temperature until the solution became colorless, after which it was re-cooled to −78 °C, and *sec*-BuLi in cyclohexane and *n*-hexane (0.246 mL, 0.300 mmol) was added to initiate the reaction. DPE (0.263 mL, 1.50 mmol) was added and the deep red solution was stirred for 30 min. A mixture of GMA (0.728 mL, 5.53 mmol) and MMA (2.36 mL, 22.2 mmol) was then added and the solution was stirred for 30 min. The solution became colorless. Degassed methanol (10 mL) was added to the flask to prepare proton-terminated PGM9-22. The polymer was precipitated in isopropyl alcohol, filtered, and dried under reduced pressure at 40 °C to obtain a white powder (2.45 g, 82% yield). ^{1}H NMR (400 MHz, $CDCl_3$, δ, ppm): 0.50–1.17 (α-CH_3, PGMA; α-CH_3, PMMA), 1.70–2.10 (-CH_2-C(CH_3)-, PGMA; -CH_2-C(CH_3)-, PMMA), 2.64 (-CH_2-CH(CH_2)-O-, PGMA), 2.85 (-CH_2-CH(CH_2)-O-, PGMA), 3.21 (-CH_2-CH(CH_2)-O-, PGMA), 3.59 (-O-CH_3, PMMA), 3.73–3.85 (-CH_2-CH(CH_2)-O-, PGMA), 4.24–4.38 (-CH_2-CH(CH_2)-O-, PGMA), 7.09–7.24 (aromatic, DPE). ^{13}C NMR (100 MHz, $CDCl_3$, δ, ppm): 16.5, 18.7, 44.6, 44.7, 44.9, 45.0, 48.0, 48.2, 48.4, 49.3, 49.5, 49.7, 51.2, 51.4, 51.6, 52.1, 52.3, 52.5, 54.2, 65.6, 176.3, 177.0, 177.1, 177.5, 177.8, 178.1. IR (KBr, v, cm^{-1}): 3447, 2996, 2951, 2843, 1730, 1636, 1485, 1448, 1436, 1388, 1367, 1338, 1270, 1243, 1192, 1150, 1064, 991, 967, 910, 845, 750, 703.

Synthesis of $PGMA_{Ph}$ by the Thiol−Epoxy Reaction

A series of PGMA derivatives was synthesized using the following procedure. A reaction vessel was charged with the PGMA homopolymer (0.303 g, 0.0240 mmol), benzenethiol (0.323 mL, 3.17 mmol; 1.5 mole equiv. of the PGMA repeating units, >98.0%), and 1 wt% aqueous $LiOH \cdot H_2O$ solution (0.453 g, 0.108 mmol; 0.05 mole equiv. of the PGMA repeating units). These reactants were dissolved in THF (6 mL; 2 mL per 100 mg of PGMA) at ambient temperature. The resulting mixture was then stirred at 40 °C for 3 h. The polymer was precipitated from a MeOH/water mixture, and the solvent was decanted to collect the precipitate. The solid was then dissolved in THF and precipitated from *n*-hexane and diethyl ether (2 times each) to remove residual thiol. Finally, the solid was dissolved in THF, precipitated with *n*-hexane, and filtered. The resulting solid was dried under reduced pressure at room temperature to obtain a white powder (0.370 g, 69% yield). ^1H NMR (400 MHz, $CDCl_3$, δ, ppm): 0.47–1.29 (α-CH_3, $PGMA_{Ph}$), 1.55–2.18 (-CH_2-C(CH_3)-, $PGMA_{Ph}$), 3.01 (-CH(OH)-CH_2-S-, $PGMA_{Ph}$), 3.80–4.23 (-(C=O)O-CH_2-C*H*(OH)-, $PGMA_{Ph}$), 7.11–7.40 (aromatic, $PGMA_{Ph}$). ^{13}C NMR (100 MHz, $CDCl_3$, δ, ppm): 18.1, 20.0, 37.5, 45.1, 53.2, 67.8, 126.9, 129.3, 130.0, 135.0, 176.9, 177.4. IR (KBr, ν, cm^{-1}): 3448, 3073, 3057, 2985, 2940, 2890, 1951, 1867, 1729, 1583, 1481, 1439, 1404, 1332, 1271, 1243, 1153, 1103, 1086, 1069, 1025, 993, 967, 932, 896, 741, 691, 613, 544, 471.

Characterization of $PGMA_{C2Ph}$

Yield: 0.438 g, 74%. ^1H NMR (400 MHz, $CDCl_3$, δ, ppm): 0.49–1.28 (α-CH_3, $PGMA_{C2Ph}$), 1.39–2.19 (-CH_2-C(CH_3)-, $PGMA_{C2Ph}$), 2.61 (-CH(OH)-CH_2-S-CH_2-CH_2-, $PGMA_{C2Ph}$), 2.71–2.97 (-CH(OH)-CH_2-S-CH_2-CH_2-, $PGMA_{C2Ph}$), 3.56 (-O*H*, $PGMA_{C2Ph}$), 3.80–4.28 (-(C=O)O-CH_2-C*H*(OH)-, $PGMA_{C2Ph}$), 7.06–7.32 (aromatic, $PGMA_{C2Ph}$). ^{13}C NMR (100 MHz, $CDCl_3$, δ, ppm): 18.0, 19.7, 34.2, 35.9, 36.3, 45.0, 45.2, 53.2, 68.1, 126.6, 128.6, 140.2, 176.9, 177.4, 177.6. IR (KBr, ν, cm^{-1}): 3448, 3105, 3085, 3061, 3026, 2996, 2921, 2852, 2647, 1947, 1883, 1806, 1728, 1603, 1584, 1496, 1487, 1453, 1404, 1389, 1365, 1329, 1272, 1243, 1176, 1153, 1107, 1073, 1030, 994, 967, 933, 898, 844, 826, 748, 715, 698, 662, 617, 565, 493.

Characterization of $PGMA_{Cy}$

Yield: 0.261 g, 48%. ^1H NMR (400 MHz, $CDCl_3$, δ, ppm): 0.51–1.17 (α-CH_3, $PGMA_{Cy}$), 1.18–2.26 (-CH_2-C(CH_3)-, $PGMA_{Cy}$; -S-CH(CH_2-CH_2)-CH_2-CH_2-CH_2-, $PGMA_{Cy}$), 2.70 (-CH(OH)-CH_2-S-C*H*(CH_2-CH_2)-CH_2-CH_2-CH_2-, $PGMA_{Cy}$), 3.66 (-O*H*, $PGMA_{Cy}$), 3.85–4.32 (-(C=O)O-CH_2-C*H*(OH)-, $PGMA_{Cy}$), 7.10–7.23 (aromatic, DPE). ^{13}C NMR (100 MHz, $CDCl_3$, δ, ppm): 17.9, 19.6, 25.8, 26.1, 33.8, 44.1, 45.0, 45.2, 53.1, 68.0, 68.4, 177.0, 177.4, 177.6. IR (KBr, ν, cm^{-1}): 3453, 2928, 2852, 2666, 1731, 1634, 1484, 1448, 1390, 1340, 1264, 1152, 1079, 998, 933, 887, 820, 746, 697, 611, 545, 521.

Characterization of PG$_F$M9-22

Yield: 0.222 g, 90%. ^{1}H NMR (400 MHz, CDCl$_3$, δ, ppm): 0.51–1.31 (α-C*H*$_3$, PGMA$_F$; α-C*H*$_3$, PMMA), 1.67–2.22 (-C*H*$_2$-C(CH$_3$)-, PGMA$_F$; -C*H*$_2$-C(CH$_3$)-, PMMA), 2.84 (-CH(OH)-C*H*$_2$-S-, PGMA$_F$), 3.16–3.38 (-S-C*H*$_2$-CF$_3$, PGMA$_F$), 3.59 (-O-C*H*$_3$, PMMA), 4.06 (-(C=O)O-C*H*$_2$-C*H*(OH)-, PGMA$_F$), 7.09–7.24 (aromatic, DPE). ^{13}C NMR (100 MHz, CDCl$_3$, δ, ppm): 16.5, 17.4, 18.9, 34.3, 34.6, 34.9, 35.2, 36.2, 44.6, 44.9, 45.1, 52.0, 54.0, 54.3, 67.8, 69.0, 124.6, 127.4, 176.8, 177.1, 177.9, 178.2. IR (KBr, ν, cm^{-1}): 3510, 2998, 2952, 2844, 1730, 1631, 1487, 1449, 1437, 1388, 1314, 1273, 1245, 1194, 1152, 1124, 1085, 989, 963, 913, 841, 750, 708, 638.

Characterization of PS-*b*-PG$_H$M10-22

Yield: 0.279 g, 88%. ^{1}H NMR (400 MHz, CDCl$_3$, δ, ppm): 0.53–1.19 (α-C*H*$_3$, PGMA$_H$; α-C*H*$_3$, PMMA), 1.21–1.66 (-S-CH$_2$-C*H*$_3$, PGMA$_H$; -C*H*$_2$-CH-, PS), 1.69–2.30 (-CH$_2$-C*H*-, PS; -C*H*$_2$-C(CH$_3$)-, PGMA$_H$; -C*H*$_2$-C(CH$_3$)-, PMMA), 2.53–2.80 (-CH(OH)-C*H*$_2$-S-C*H*$_2$-CH$_3$, PGMA$_H$), 3.60 (-O-C*H*$_3$, PMMA), 3.91–4.21 (-(C=O)O-C*H*$_2$-C*H*(OH)-, PGMA$_H$), 6.21–6.85 (*o*-aromatic, PS), 6.85–7.24 (*m*, *p*-aromatic, PS). ^{13}C NMR (100 MHz, CDCl$_3$, δ, ppm): 14.9, 16.5, 17.2, 18.8, 26.6, 30.4, 35.3, 40.4, 40.6, 44.6, 45.0, 51.9, 54.4, 68.0, 125.6, 125.7, 127.4, 127.5, 127.7, 128.0, 128.3, 145.2, 145.4, 145.7, 177.0, 177.9, 178.2. IR (KBr, ν, cm^{-1}): 3481, 3103, 3082, 3060, 3026, 2999, 2948, 2925, 2849, 1943, 1863, 1803, 1731, 1635, 1602, 1493, 1453, 1434, 1388, 1271, 1242, 1192, 1150, 1071, 1031, 987, 967, 907, 846, 757, 699, 540.

Characterization of PS-*b*-PG$_{Ph}$M10-22

Yield: 0.289 g, 87%. ^{1}H NMR (400 MHz, CDCl$_3$, δ, ppm): 0.54–1.19 (α-C*H*$_3$, PGMA$_{Ph}$; α-C*H*$_3$, PMMA), 1.43 (-C*H*$_2$-CH-, PS), 1.68–2.30 (-CH$_2$-C*H*-, PS; -C*H*$_2$-C(CH$_3$)-, PGMA$_{Ph}$; -C*H*$_2$-C(CH$_3$)-, PMMA), 3.08 (-CH(OH)-C*H*$_2$-S-, PGMA$_{Ph}$), 3.60 (-O-C*H*$_3$, PMMA), 3.89–4.25 (-(C=O)O-C*H*$_2$-C*H*(OH)-, PGMA$_{Ph}$), 6.30–6.86 (*o*-aromatic, PS), 6.86–7.52 (*m*, *p*-aromatic, PS; aromatic, PGMA$_{Ph}$). ^{13}C NMR (100 MHz, CDCl$_3$, δ, ppm): 16.5, 17.3, 40.5, 44.6, 44.9, 51.5, 52.4, 54.3, 67.9, 125.1, 126.3, 127.1, 127.4, 128.4, 128.7, 129.7, 130.3, 135.2, 145.2, 145.4, 145.7, 146.2, 177.1, 177.9, 178.2. IR (KBr, ν, cm^{-1}): 3480, 3107, 3080, 3057, 3025, 3001, 2948, 2925, 2848, 1947, 1867, 1806, 1731, 1635, 1601, 1586, 1493, 1483, 1453, 1440, 1385, 1366, 1272, 1242, 1192, 1150, 1084, 1065, 1027, 985, 967, 905, 845, 751, 699, 537.

Characterization of PS-*b*-PG$_{C2Ph}$M10-22

Yield: 0.288 g, 85%. ^{1}H NMR (400 MHz, CDCl$_3$, δ, ppm): 0.53–1.18 (α-C*H*$_3$, PGMA$_{C2Ph}$; α-C*H*$_3$, PMMA), 1.43 (-C*H*$_2$-CH-, PS), 1.67–2.29 (-CH$_2$-C*H*-, PS; -C*H*$_2$-C(CH$_3$)-, PGMA$_{C2Ph}$; -C*H*$_2$-C(CH$_3$)-, PMMA), 2.68(-CH(OH)-C*H*$_2$-S-CH$_2$-CH$_2$-, PGMA$_{C2Ph}$), 2.76–2.97 (-CH(OH)-CH$_2$-S-C*H*$_2$-C*H*$_2$-, PGMA$_{C2Ph}$), 3.59 (-O-C*H*$_3$, PMMA), 3.88–4.18 (-(C=O)O-C*H*$_2$-C*H*(OH)-, PGMA$_{C2Ph}$), 6.27–6.86 (*o*-aromatic, PS), 6.86–7.37 (*m*, *p*-aromatic, PS; aromatic, PGMA$_{C2Ph}$). ^{13}C NMR (100 MHz, CDCl$_3$, δ, ppm): 16.6, 17.2, 34.3, 35.9, 36.3, 40.4, 40.7, 44.7, 45.0, 52.0, 54.3, 68.1, 125.6, 125.7, 126.6, 127.4, 127.5, 127.7, 128.1, 128.3, 128.6, 140.2, 145.4, 145.8, 146.2, 177.2, 177.9, 178.2. IR (KBr, ν, cm^{-1}): 3503, 3103, 3083, 3060, 3026, 3000, 2947, 2925, 2849, 1943, 1871, 1806, 1731, 1639, 1602, 1493, 1453, 1434, 1387, 1366, 1271, 1242, 1192, 1150, 1071, 1031, 988, 967, 909, 837, 755, 699, 539.

Characterization of PS-*b*-PG$_{Cy}$M10-22

Yield: 0.293 g, 88%. ^{1}H NMR (400 MHz, CDCl$_3$, δ, ppm): 0.54–1.18 (α-CH_3, PGMA$_{Cy}$; α-CH_3, PMMA), 1.19–2.28 (-CH_2-CH-, PS; -CH_2-C(CH$_3$)-, PGMA$_{Cy}$; -S-CH(CH_2-CH_2)-CH_2-CH_2-CH_2-, PGMA$_{Cy}$; -CH_2-C(CH$_3$)-, PMMA), 2.58–2.82 (-CH(OH)-CH_2-S-CH(CH$_2$-CH$_2$)-CH$_2$-CH$_2$-CH$_2$-, PGMA$_{Cy}$), 3.60 (-O-CH_3, PMMA), 3.88–4.19 (-(C=O)O-CH_2-CH(OH)-, PGMA$_{Cy}$), 6.21–6.84 (*o*-aromatic, PS), 6.84–7.24 (*m*, *p*-aromatic, PS). ^{13}C NMR (100 MHz, CDCl$_3$, δ, ppm): 16.6, 17.1, 18.8, 25.8, 26.2, 32.9, 33.8, 40.3, 44.1, 44.6, 44.8, 45.0, 50.0, 51.9, 54.3, 68.0, 68.3, 125.6, 125.7, 127.4, 127.5, 127.7, 128.0, 128.1, 128.3, 145.2, 145.4, 145.7, 146.1, 177.0, 177.9, 178.2. IR (KBr, ν, cm^{-1}): 3481, 3103, 3083, 3060, 3026, 3000, 2927, 2851, 1947, 1867, 1803, 1733, 1631, 1602, 1493, 1452, 1438, 1388, 1365, 1270, 1242, 1192, 1150, 1066, 1031, 989, 967, 905, 841, 755, 699, 539.

Synthesis of PS-*r*-PMMA-*r*-PHEMA (NL38) by Free Radical Polymerization

2,2'-Azobis(isobutyronitrile) (AIBN) (>98.0%) was purified by recrystallization from MeOH. 2-Hydroxyethyl methacrylate (HEMA) (>95.0%) was passed through an activated alumina column. AIBN (53.4 mg, 0.325 mmol), styrene (0.920 mL, 8.00 mmol), MMA (1.17 mL, 11.0 mmol), and HEMA (0.122 mL, 1.00 mmol) were added to a tube-shaped reaction vessel and degassed by bubbling with Ar for 5 min. The mixture was stirred at 80 °C for 8 h. Polymerization was quenched by rapid cooling to 10 °C. The crude product was diluted with THF, precipitated from MeOH, and filtered. The precipitate was dried under reduced pressure at 40 °C to yield PS-*r*-PMMA-*r*-PHEMA as a white powder (1.96 g, 95% yield). ^{1}H NMR (400 MHz, CDCl$_3$, δ, ppm): 0.32–1.14, 1.14–2.54, 2.63–4.21, 6.55–7.24. ^{13}C NMR (100 MHz, CDCl$_3$, δ, ppm): 16.6, 18.4, 20.0, 21.8, 38.7, 44.3, 45.5, 46.3, 51.0, 51.7, 54.4, 60.8, 126.2, 128.0, 128.3, 145.2, 146.8, 176.8, 177.5. IR (KBr, ν, cm^{-1}): 3529, 3445, 3107, 3084, 3061, 3027, 2989, 2948, 2843, 1955, 1871, 1730, 1602, 1495, 1468, 1454, 1436, 1386, 1272, 1232, 1196, 1141, 1075, 1031, 989, 967, 907, 843, 810, 803, 760, 701, 544, 484.

Determining the Densities of the Synthesized Homopolymer by Gradient column

The densities of $PGMA_{Ph}$, $PGMA_{C2Ph}$, and $PGMA_{Cy}$ were determined by placing each homopolymer into a gradient column prepared from aqueous KI (>99.5%) solution calibrated with organic reagents of known densities. The reagents used, positions on the graduated cylinder, and estimated densities were summarized in Supplementary Table 2, 3, and 4.

Determining the Surface Free Energies of the Synthesized Random Copolymers

The surface free energies (SFEs) of the PG_FM random copolymers with different $PGMA_F$ contents were calculated by measuring the contact angles (C. A.) of water and diiodomethane (>99.0%) on the random copolymer thin films.

The general procedure for preparation of random copolymer thin films is as follows. Silicon wafers were cut into 1.5×1.5 cm pieces and wiped with toluene-soaked Kim Wipes. The wafers were then sequentially sonicated in acetone, ethanol, and toluene (1 min each), dried in a stream of nitrogen, and further dried by heating to 100 °C. The random copolymer thin films with film thicknesses of 110-120 nm were prepared on the cleaned wafers by spin-coating 5 wt% PG_FM solutions in propylene glycol 1-monomethyl ether 2-acetate (PGMEA) at 2000 rpm for 60 s. These PG_FM thin films were dried under reduced pressure at 40 °C for 1 h to remove residual PGMEA.

Determining the Effective Flory–Huggins Interaction Parameter (χ_{eff})

Estimation of χ_{eff} was demonstrated based on the method described by Yoshimura et al.[1]. Molten BCP samples were placed in 0.01 mm-thick glass capillaries, and their SAXS profiles were collected every 10 °C during cooling. At each measurement temperature, the samples were held for 60 min before exposure to X-ray radiation for 30 min to collect the spectra. The background spectra were collected by exposing an empty glass capillary for 30 min at each measurement temperature. The background spectra were subtracted from the experimental spectra to remove any potential scattering from the capillary or air.

χ_{eff} was estimated using the following equations. The scattering function $I(q)$ from the disordered melt of the BCPs with a dispersity in the molecular weight and asymmetry in the segmental volume was summarized below, where A_0 is a fitting constant, $S(q)$ and $W(q)$ together are correlation functions of the BCP, $g(q)$ is a modified Debye function and $y(q)$ is a dimensionless wave vector, r_c is the degree of polymerization of the BCP normalized by molar volume, f_X is volume fraction of X (PS or PG$_F$M), v_X is the molar volume of X. N_X is the degree of polymerization normalized over a common reference volume (v_0 = 118 Å^3) for X, FW_X is the formula weight of X, N_A is Avogadro's constant, and b_X is the segmental length of X. Here we assumed the dispersities for each homopolymer segment (D_X) are equal and were estimated from the M_w/M_n of the BCP and weight fraction of X (w_X). The FW_{PGFM} and ρ_{PGFM} were calculated from the composition ratio of PG$_F$M based on the formula weights (100.12 g mol^{-1} for MMA and 258.26 g mol^{-1} for GMA$_F$) and densities (1.18 g cm^{-3} for PMMA and 1.43 g cm^{-3} for PGMA$_F$), respectively. Four parameters, including b_X for both polymers, A_0, and χ_{eff} were optimized in a least-squares fit to the SAXS profiles at a certain temperature. The parameters for each sample were summarized in Supplementary Table 7 and 8. The estimation of χ_{eff} for each sample was done at temperatures higher than the transition from mean-field to non-mean-field.

$$I(q) = \frac{A_0}{\frac{S(q)}{W(q)} - 2\chi}$$

$$S(q) = \langle S_{PS,PS} \rangle + 2\langle S_{PS,PG_FM} \rangle + \langle S_{PG_FM,PG_FM} \rangle$$

$$W(q) = \langle S_{PS,PS} \rangle \cdot \langle S_{PG_FM,PG_FM} \rangle - \langle S_{PS,PG_FM} \rangle^2$$

$$\langle S_{PS,PS}(q) \rangle = r_c {f_{PS}}^2 {g^{(2)}}_{PS}(q)$$

$$\langle S_{PG_FM,PG_FM}(q) \rangle = r_c {f_{PG_FM}}^2 {g^{(2)}}_{PG_FM}(q)$$

$$\langle S_{PS,PG_FM}(q) \rangle = r_c f_{PS} f_{PG_FM} {g^{(1)}}_{PS}(q) {g^{(1)}}_{PG_FM}(q)$$

$$r_c = \frac{(v_{PS}N_{PS} + v_{PG_FM}N_{PG_FM})}{(v_{PS} \cdot v_{PG_FM})^{\frac{1}{2}}}$$

$$v_{PS} = \frac{FW_{PS}}{\rho_{PS}N_A} \quad v_{PG_FM} = \frac{FW_{PG_FM}}{\rho_{PG_FM}N_A}$$

$${g^{(1)}}_{PS}(q) = \frac{1}{y_{PS}(q)} \cdot \left\{1 - [y_{PS}(q) \cdot (D_{PS} - 1) + 1]^{-(D_{PS}-1)^{-1}}\right\}$$

$${g^{(1)}}_{PG_FM}(q) = \frac{1}{y_{PG_FM}(q)} \cdot \left\{1 - \left[y_{PG_FM}(q) \cdot \left(D_{PG_FM} - 1\right) + 1\right]^{-\left(D_{PG_FM}-1\right)^{-1}}\right\}$$

$$g^{(2)}{}_{PS}(q) = \frac{2}{y_{PS}(q)^2} \cdot \left\{-1 + y_{PS}(q) + [y_{PS}(q) \cdot (D_{PS} - 1) + 1]^{-(D_{PS}-1)^{-1}}\right\}$$

$$g^{(2)}{}_{PG_FM}(q) = \frac{2}{y_{PG_FM}(q)^2} \cdot \left\{-1 + y_{PG_FM}(q) + \left[y_{PG_FM}(q) \cdot \left(D_{PG_FM} - 1\right) + 1\right]^{-\left(D_{PG_FM}-1\right)^{-1}}\right\}$$

$$y_{PS}(q) = \frac{N_{PS} b_{PS}{}^2}{6} q^2 \quad y_{PG_FM}(q) = \frac{N_{PG_FM} b_{PG_FM}{}^2}{6} q^2 \quad D \equiv D_{PS} = D_{PG_FM} = \frac{M_w/M_n - 1}{w_{PS}{}^2 + w_{PG_FM}{}^2} + 1$$

$$N_{PS} = \frac{v_{PS} M_{n,\,PS}}{v_0 F W_{PS}} \quad N_{PG_FM} = \frac{v_{PG_FM} M_{n,\,PG_FM}}{v_0 F W_{PG_FM}}$$

Supplementary Discussion

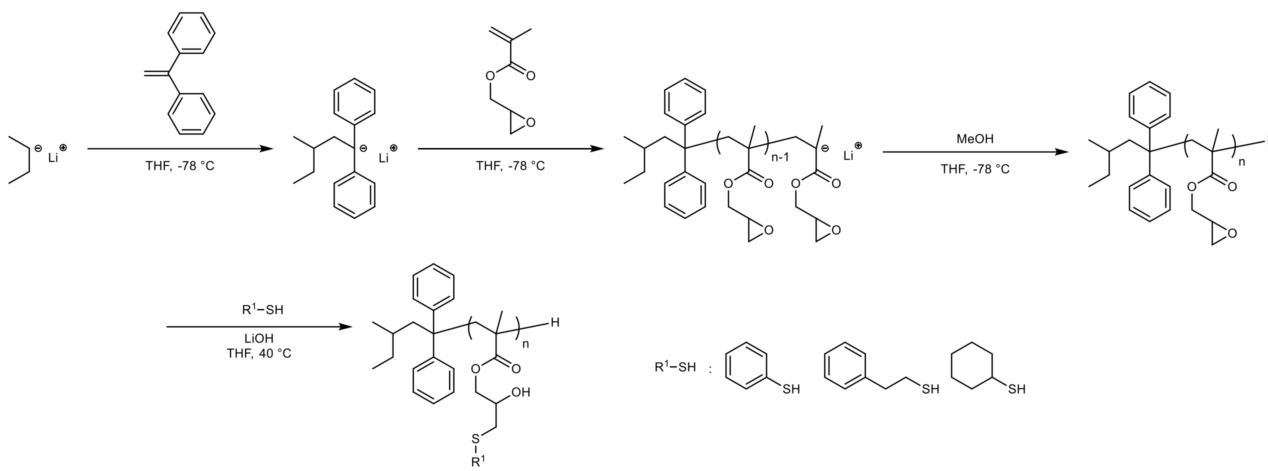

Supplementary Figure 1 | Scheme depicting the synthesis of poly(glycidyl methacrylate) (PGMA) and its derivatives by living anionic polymerization and post-functionalization involving the thiol–epoxy reaction.

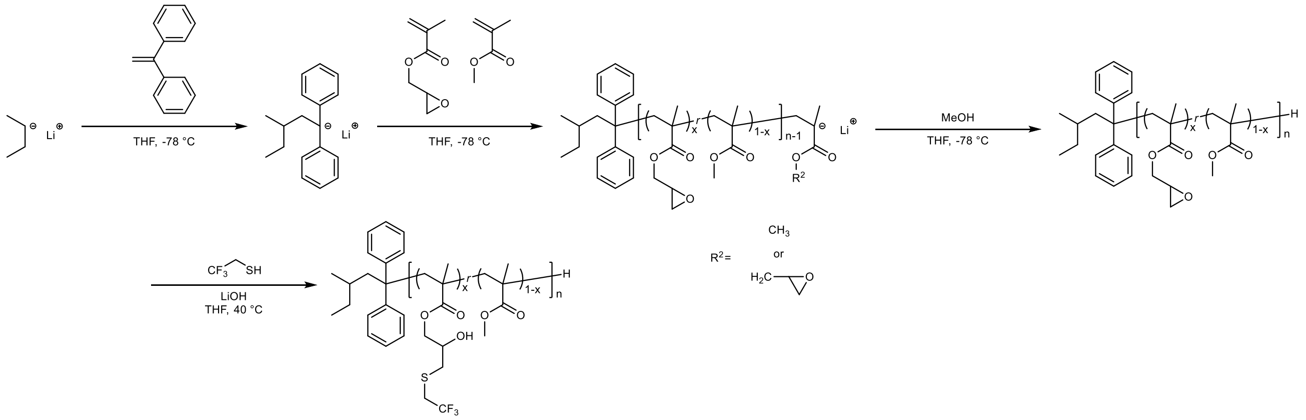

Supplementary Figure 2 | Scheme depicting the synthesis of poly(glycidyl methacrylate)-*random*-poly(methyl methacrylate) (PGM) and its derivative (PG$_F$M) by living anionic polymerization and post-functionalization involving the thiol–epoxy reaction.

Supplementary Table 1 | Characterization data for synthesized PGMA, PGMs, and their derivatives

Sample[a]	Thiol	M_n[b] (kg mol^{-1})	$Đ$[b]	PGMA[c] (mol%)	Conv. of PGMA[c]
PGMA	–	12.6	1.06	100	–
$PGMA_{Ph}$	Benzenethiol	17.1	1.07	100	>99%
$PGMA_{C2Ph}$	2-Phenylethanethiol	19.4	1.07	100	>99%
$PGMA_{Cy}$	Cyclohexanethiol	19.7	1.05	100	>99%
PGM9-33	–	9.34	1.04	33	–
PGM9-22	–	9.27	1.05	22	–
PGM9-12	–	9.16	1.04	12	–
PG_FM9-33	2,2,2-Trifluoroethanethiol	14.4	1.03	33	>99%
PG_FM9-22	2,2,2-Trifluoroethanethiol	10.7	1.04	22	>99%
PG_FM9-12	2,2,2-Trifluoroethanethiol	10.9	1.04	12	>99%

[a]PGM*X*-*Y* refers to a random copolymer with $M_n = X$ kg mol^{-1} and with *Y* mol% PGMA units and PG_FM*X*-*Y* was synthesized from the corresponding PGM*X*-*Y*.

[b]Determined by SEC in THF against PS standards.

[c]Determined by ^{1}H NMR spectroscopy in $CDCl_3$.

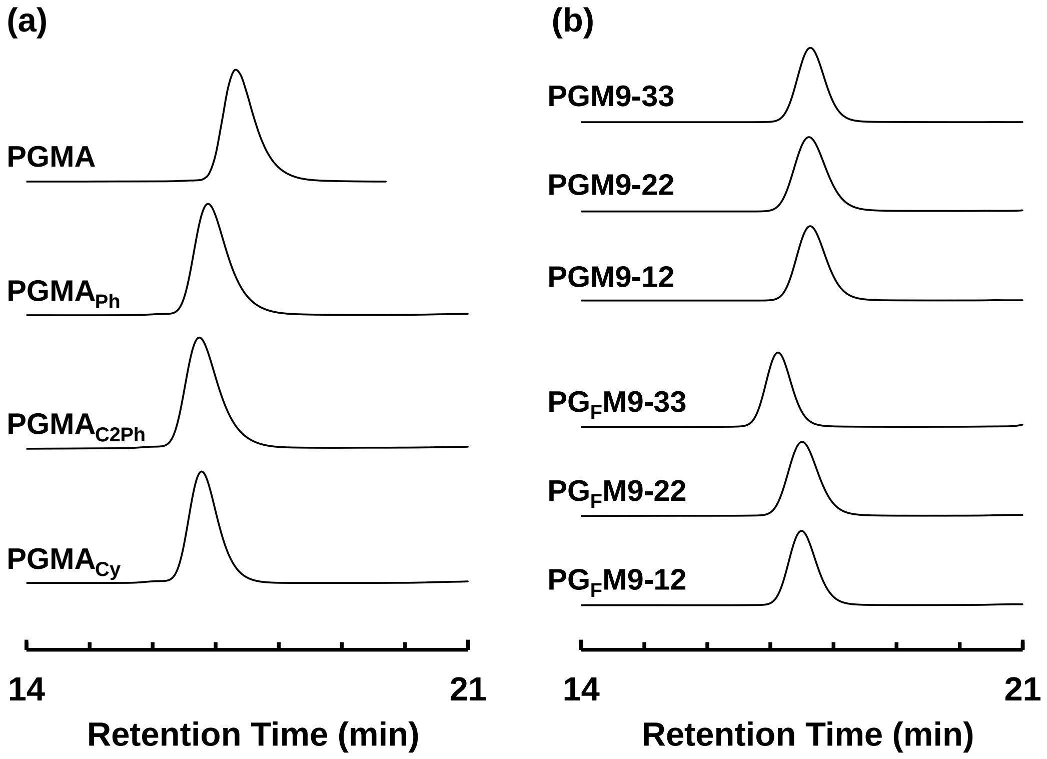

Supplementary Figure 3 | SEC traces of the (a) PGMA homopolymer and its derivatives and (b) PGMs and PG_FMs in THF.

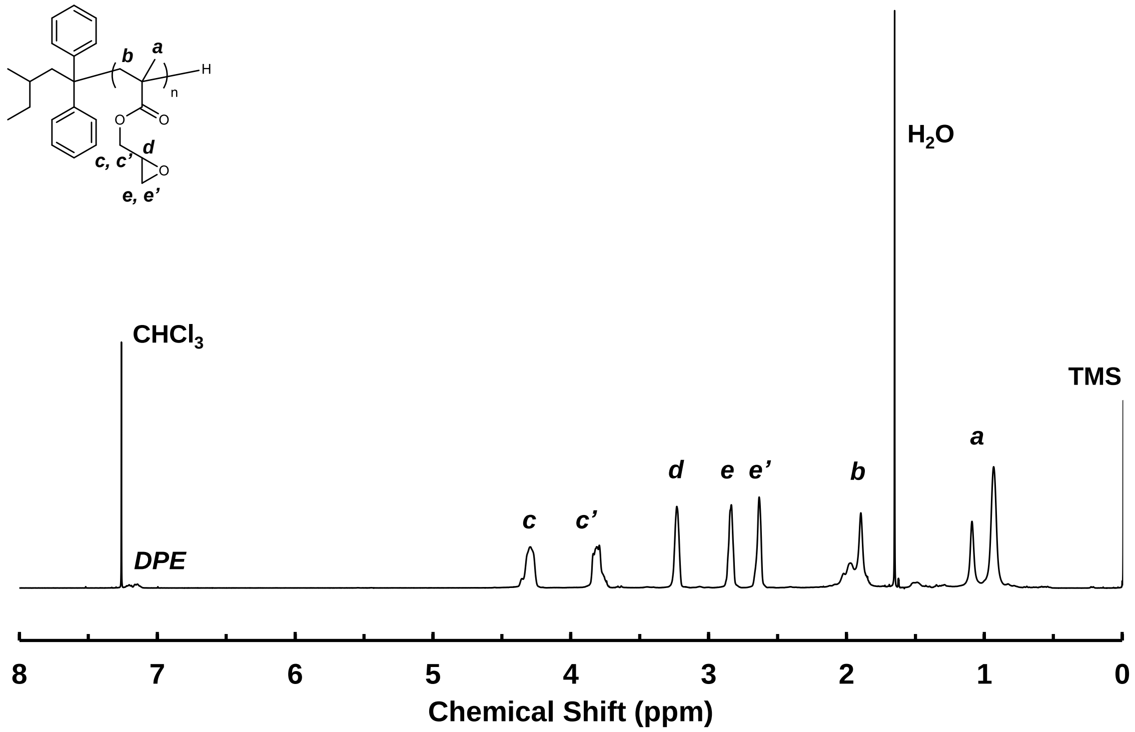

Supplementary Figure 4 | ^{1}H NMR spectrum of PGMA homopolymer in $CDCl_3$.

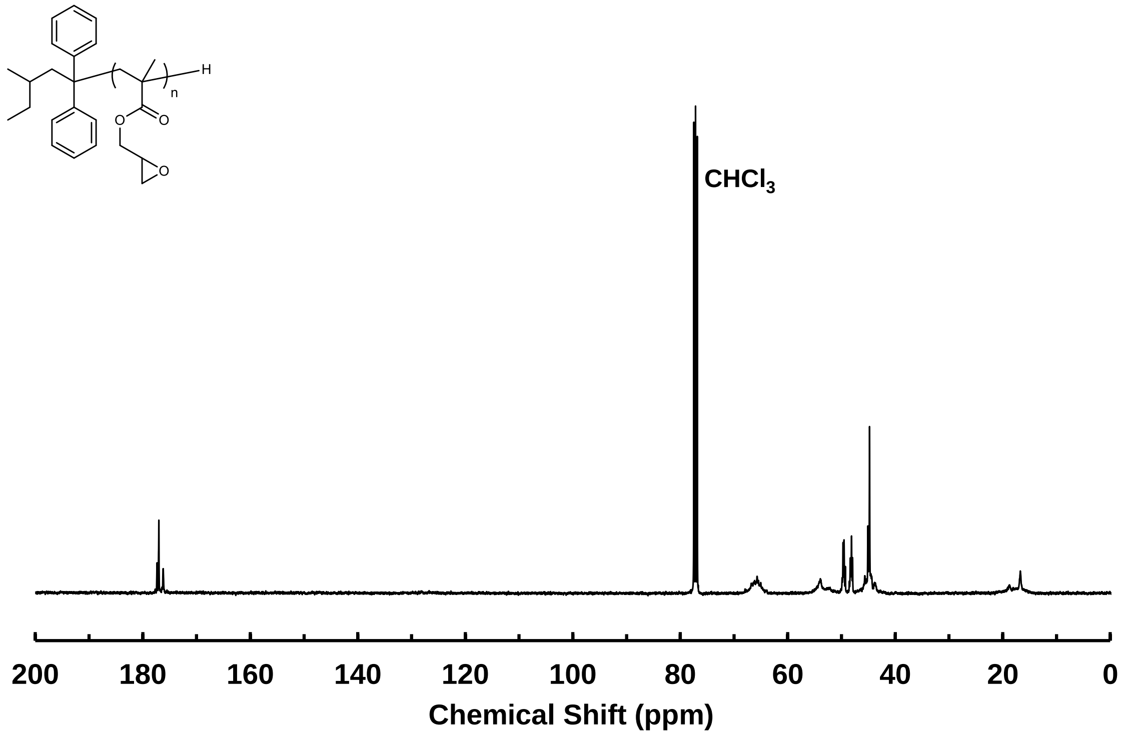

Supplementary Figure 5 | ^{13}C NMR spectrum of PGMA homopolymer in $CDCl_3$.

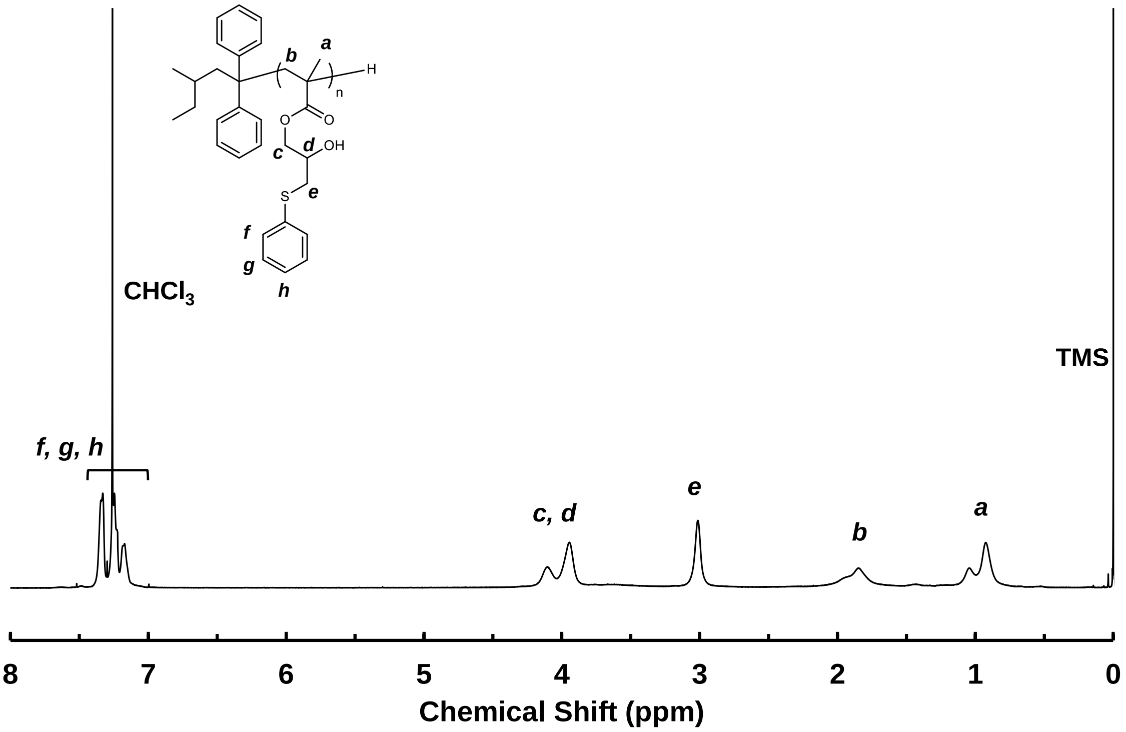

Supplementary Figure 6 | ^{1}H NMR spectrum of $PGMA_{Ph}$ homopolymer in $CDCl_3$.

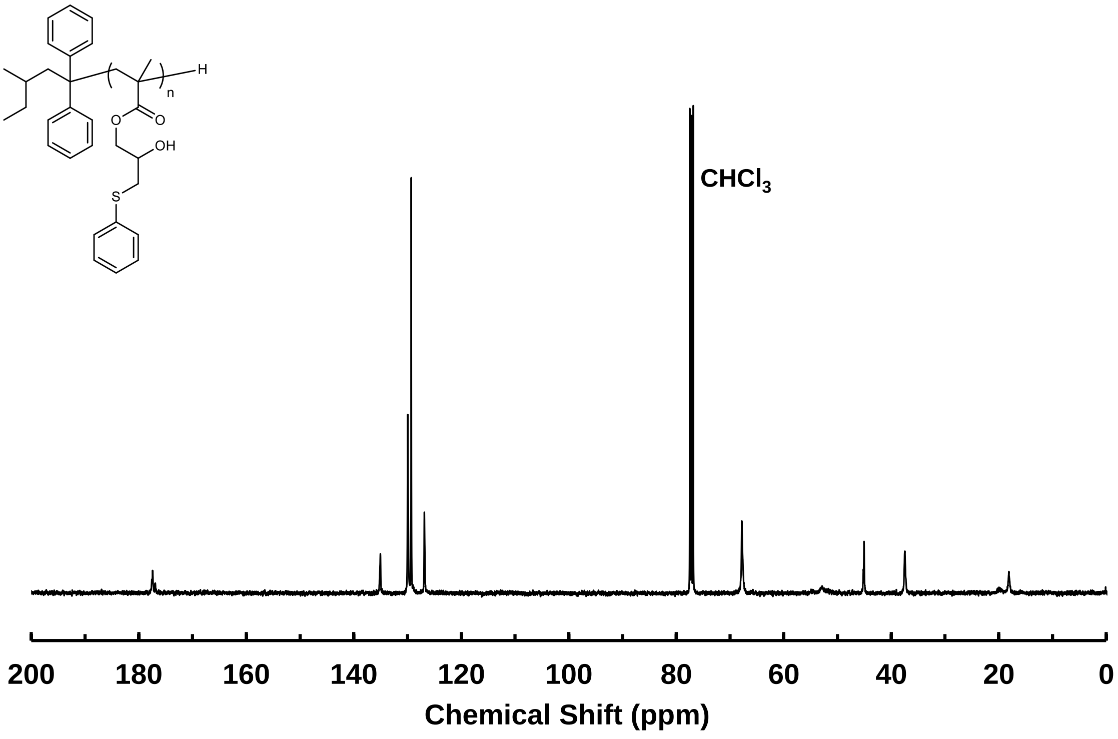

Supplementary Figure 7 | ^{13}C NMR spectrum of $PGMA_{Ph}$ homopolymer in $CDCl_3$.

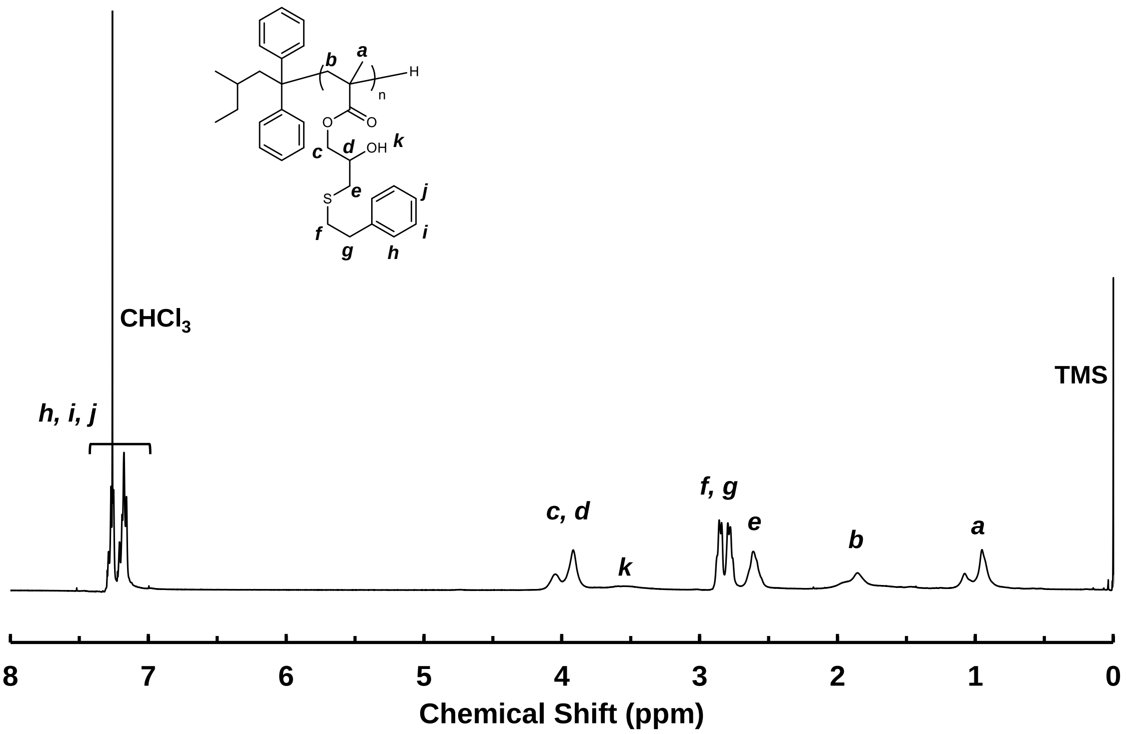

Supplementary Figure 8 | ^{1}H NMR spectrum of $PGMA_{C2Ph}$ homopolymer in $CDCl_3$.

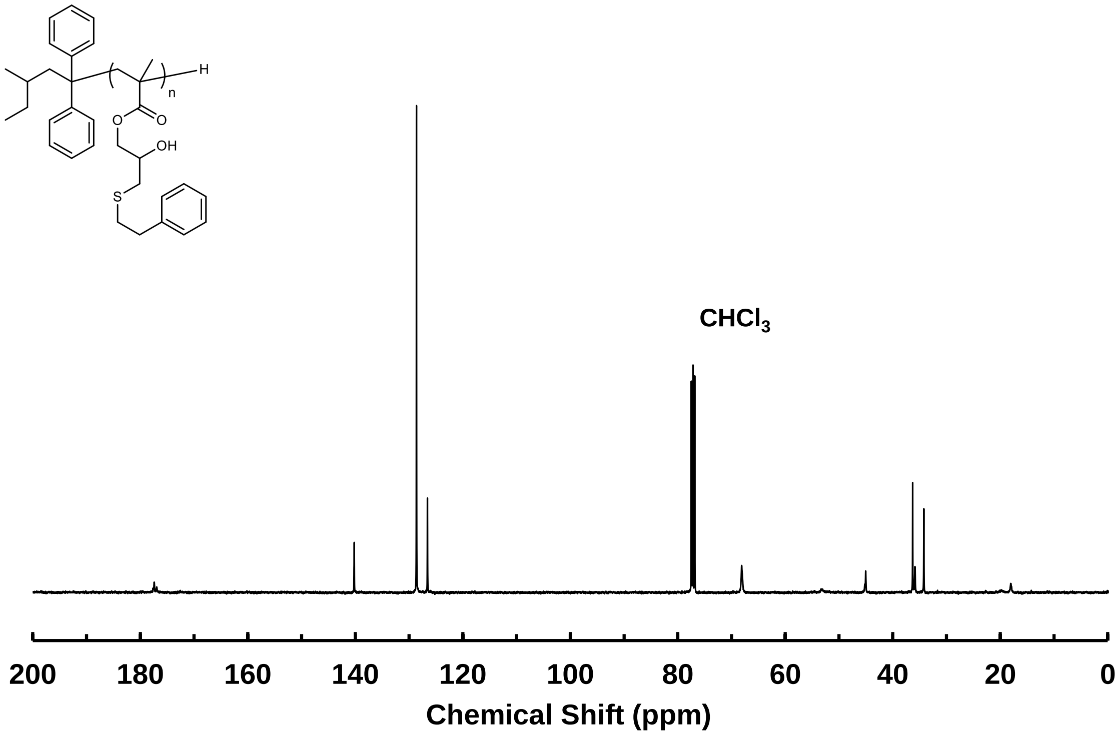

Supplementary Figure 9 | ^{13}C NMR spectrum of $PGMA_{C2Ph}$ homopolymer in $CDCl_3$.

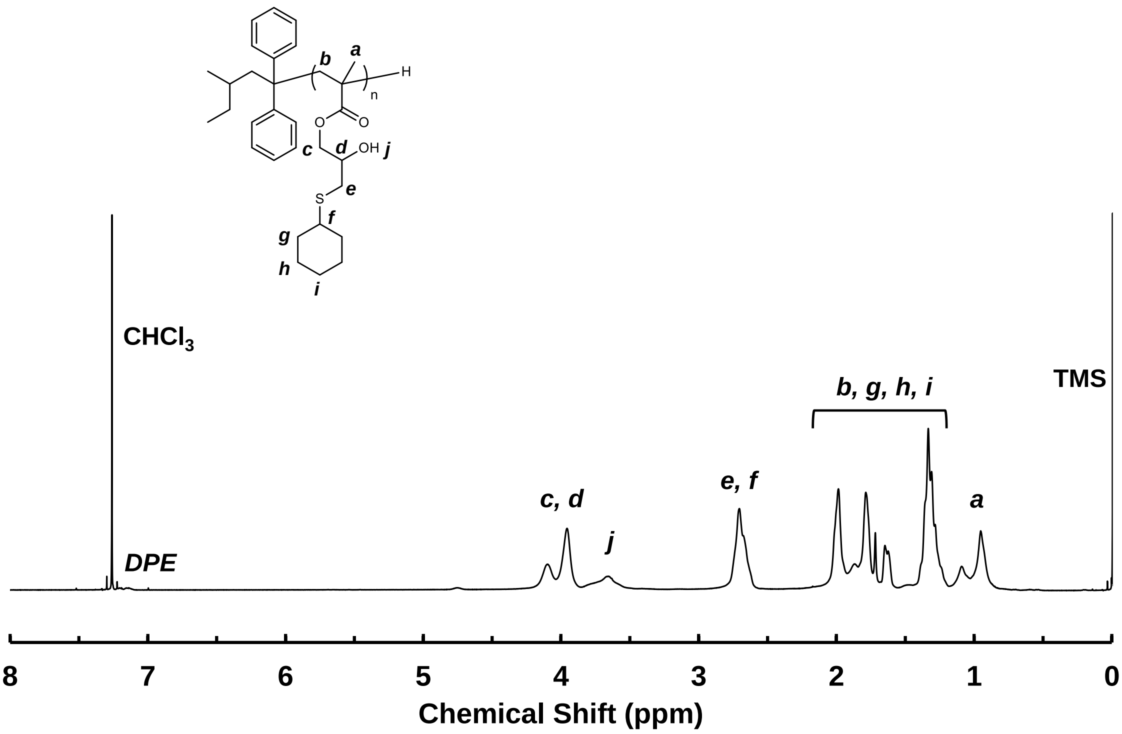

Supplementary Figure 10 | ^{1}H NMR spectrum of $PGMA_{Cy}$ homopolymer in $CDCl_3$.

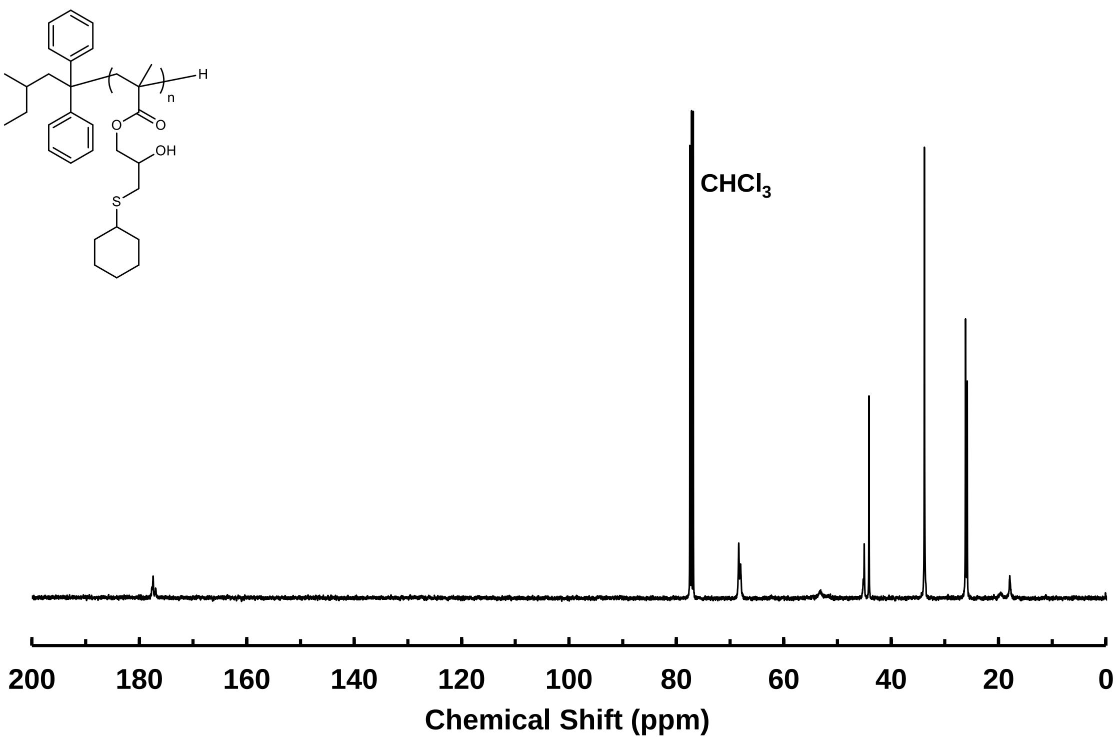

Supplementary Figure 11 | ^{13}C NMR spectrum of $PGMA_{Cy}$ homopolymer in $CDCl_3$.

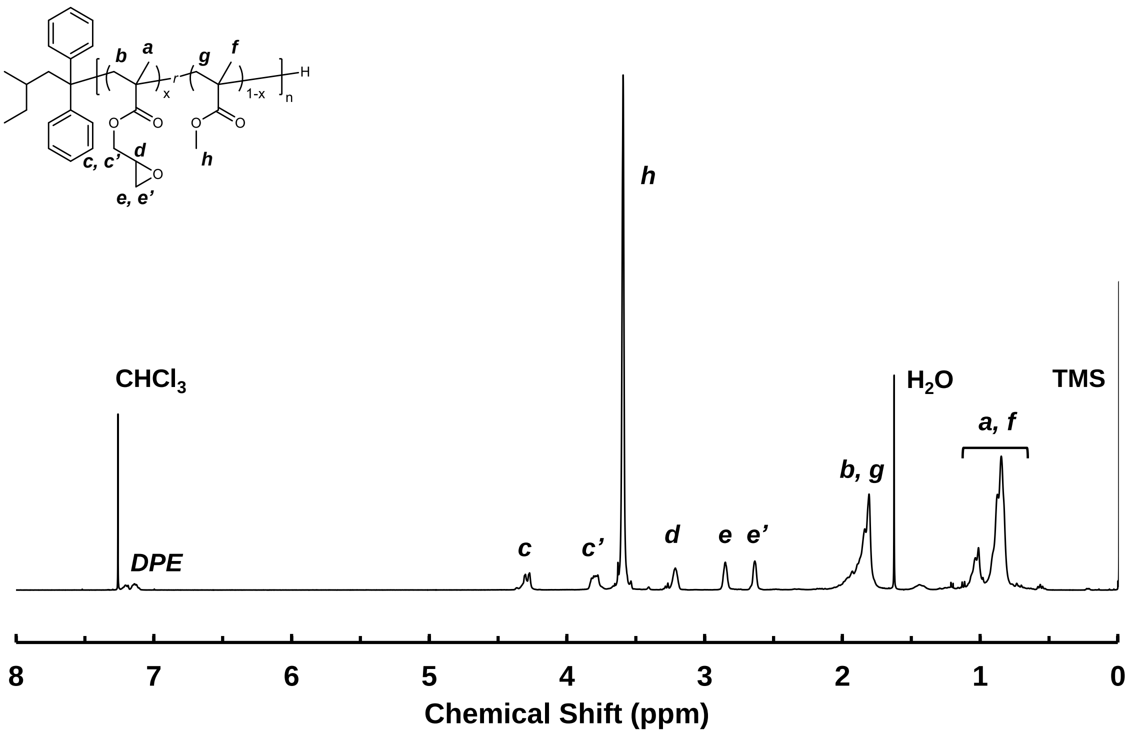

Supplementary Figure 12 | ^{1}H NMR spectrum of PGM9-22 random copolymer in $CDCl_3$.

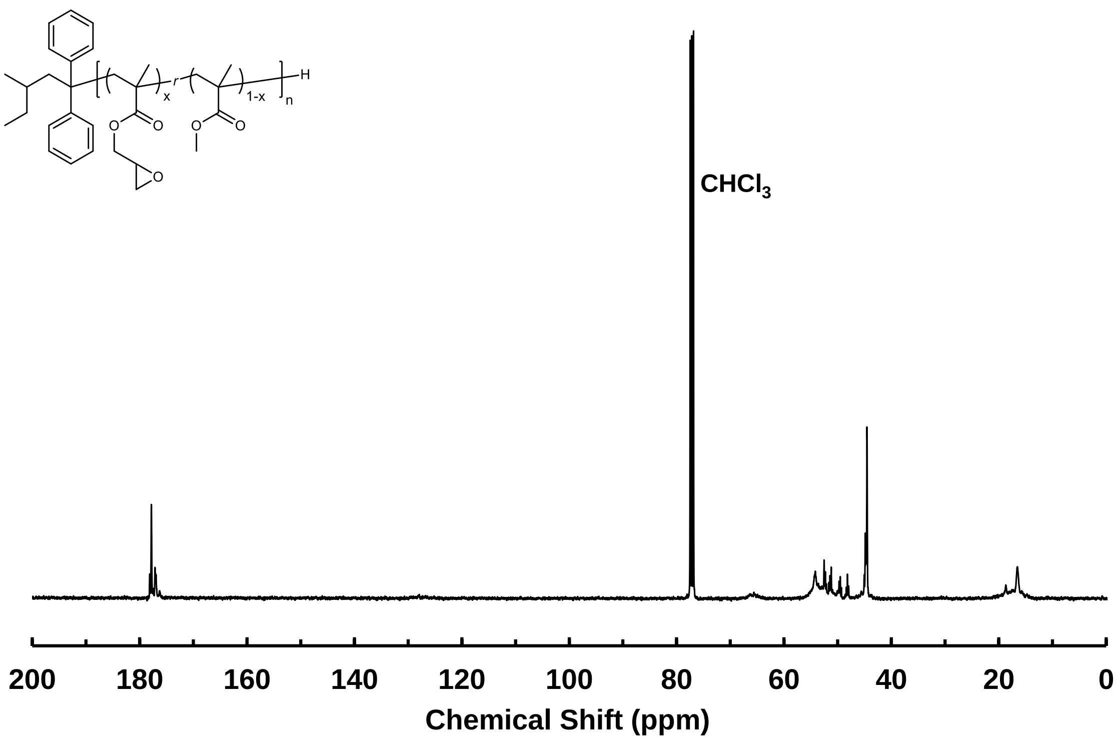

Supplementary Figure 13 | ^{13}C NMR spectrum of PGM9-22 random copolymer in $CDCl_3$.

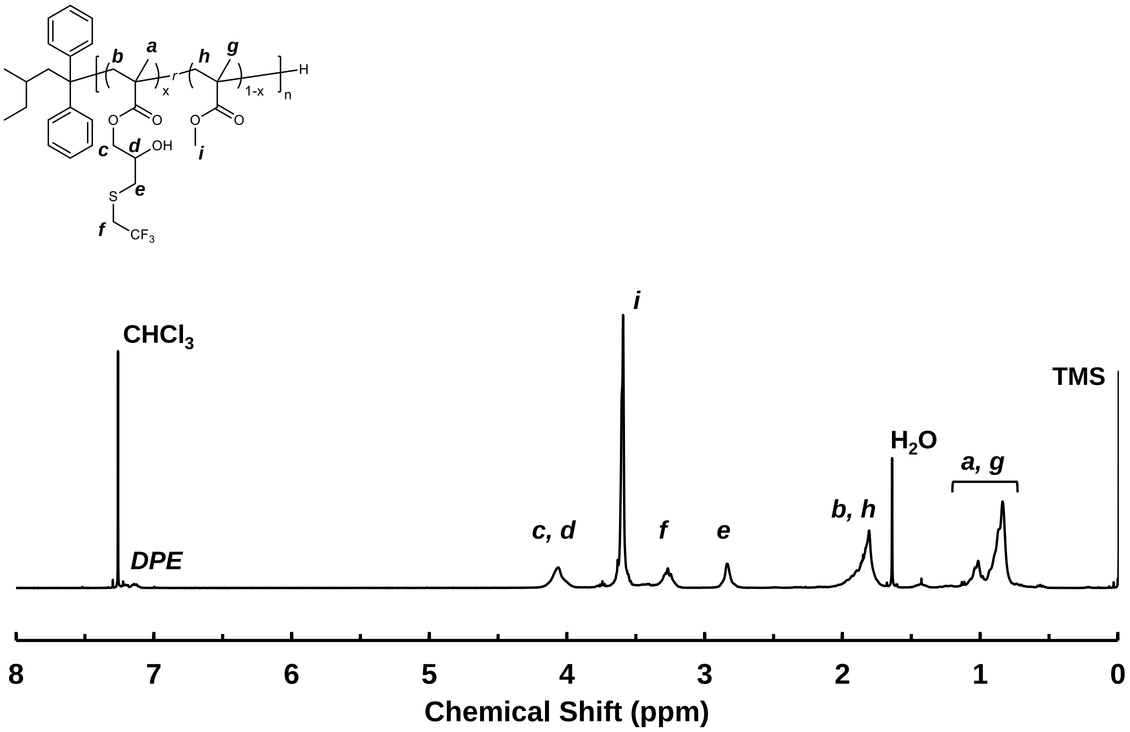

Supplementary Figure 14 | ^{1}H NMR spectrum of PG$_F$M9-22 random copolymer in $CDCl_3$.

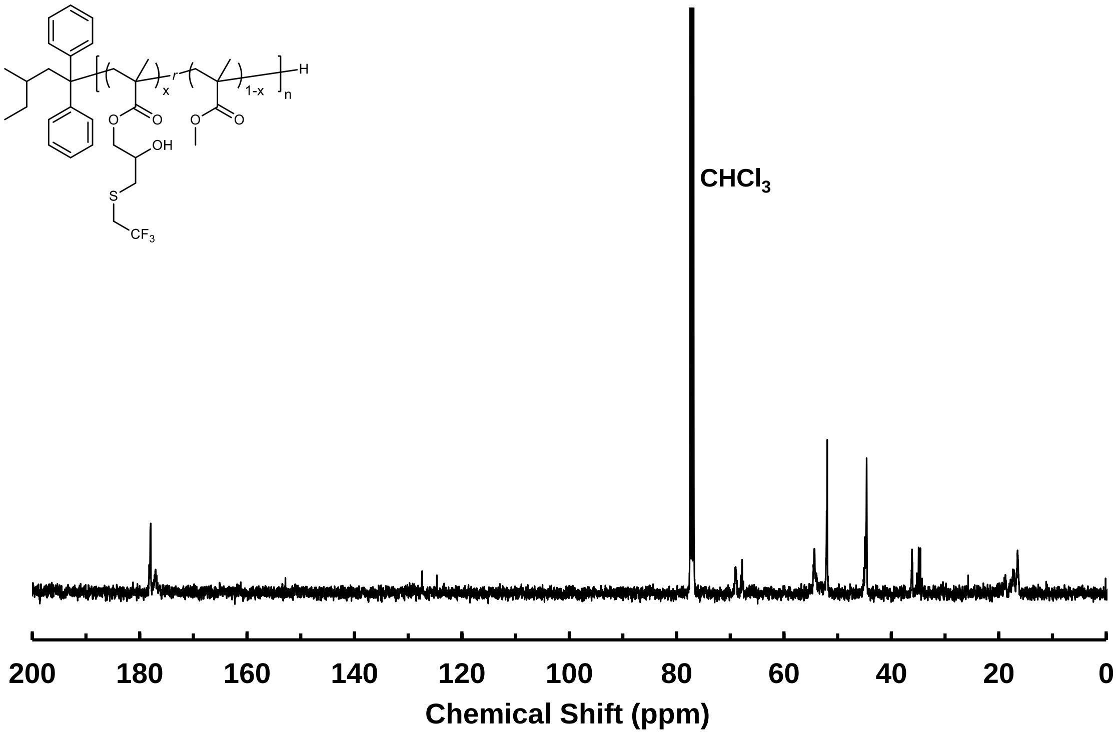

Supplementary Figure 15 | ^{13}C NMR spectrum of PG$_F$M9-22 random copolymer in $CDCl_3$.

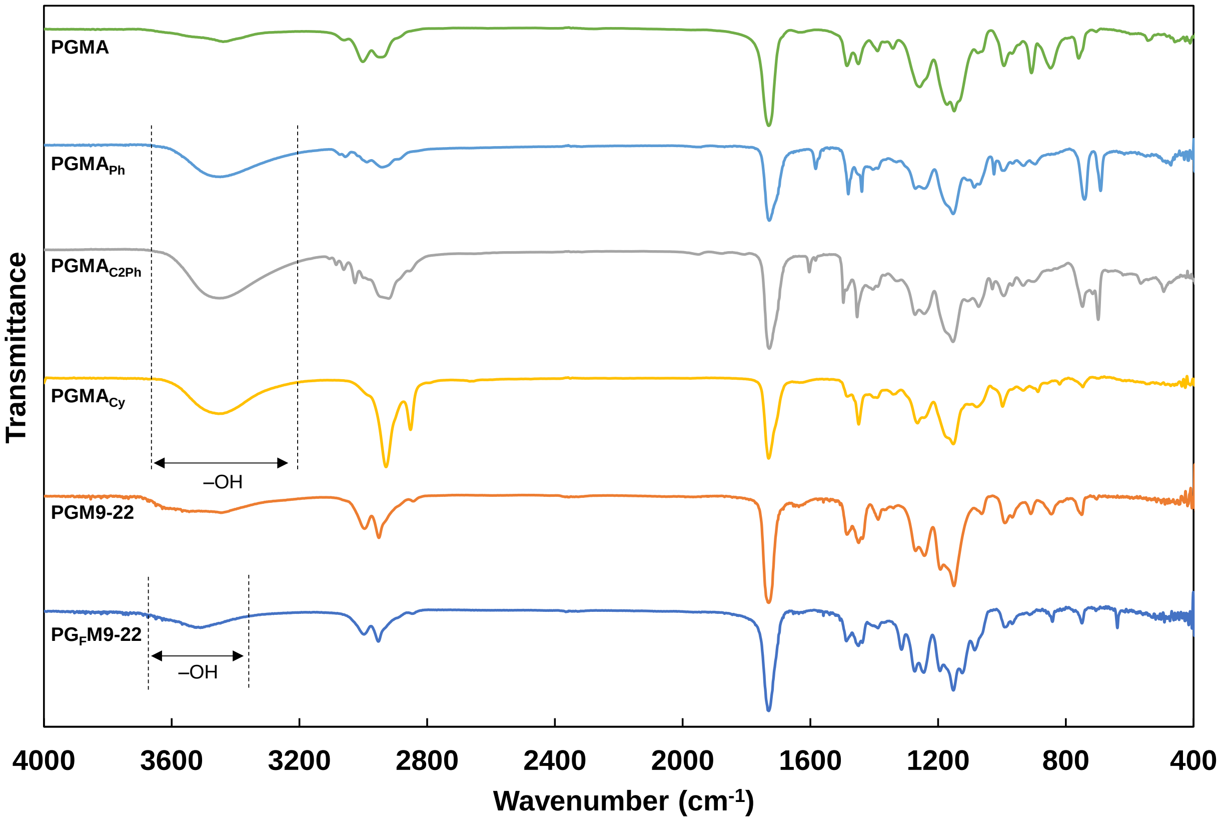

Supplementary Figure 16 | FT-IR spectra of the synthesized PGMA, PGM9-22, and their derivatives.

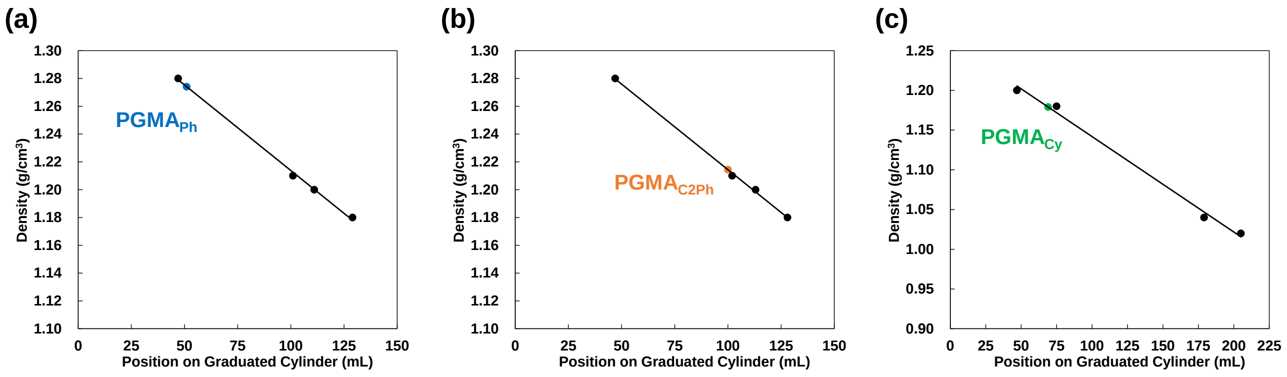

Supplementary Figure 17 | Determining the densities of the (a) $PGMA_{Ph}$, (b) $PGMA_{C2Ph}$, and (c) $PGMA_{Cy}$ by gradient column.

Supplementary Table 2 | The Positions on the graduated cylinder and densities of the organic reagents for the calibration of gradient column to determine the density of $PGMA_{Ph}$

Reagent	Position on Graduated Cylinder (mL)	Density (g cm^{-3})
1-Bromohexane	129	1.18
Nitrobenzene	111	1.20
6-Bromo-1-hexene	101	1.21
$PGMA_{Ph}$	51	1.27
1-Bromobutane	47	1.28

Supplementary Table 3 | The positions on the graduated cylinder and densities of the organic reagents for the calibration of gradient column to determine the density of $PGMA_{C2Ph}$

Reagent	Position on Graduated Cylinder (mL)	Density (g cm^{-3})
1-Bromohexane	128	1.18
Nitrobenzene	113	1.20
6-Bromo-1-hexene	102	1.21
$PGMA_{C2Ph}$	100	1.21
1-Bromobutane	47	1.28

Supplementary Table 4 | The positions on the graduated cylinder and densities of the organic reagents for the calibration of gradient column to determine the density of $PGMA_{Cy}$

Reagent	Position on Graduated Cylinder (mL)	Density (g cm^{-3})
1-Bromotetradecane	205	1.02
1-Bromododecane	179	1.04
1-Bromohexane	75	1.18
$PGMA_{Cy}$	69	1.18
Nitrobenzene	47	1.20

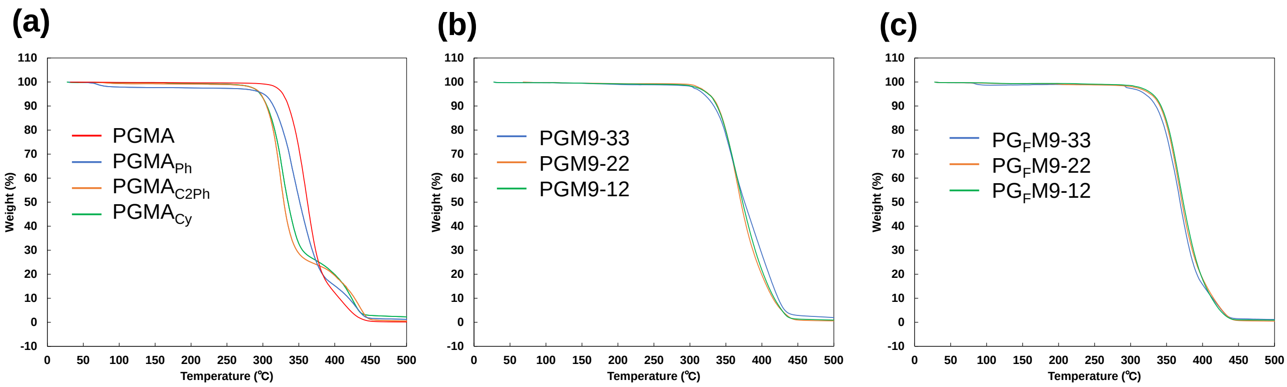

Supplementary Figure 18 | TG curves of the (a) PGMA and its derivatives, (b) PGMs, and (c) PG_FMs.

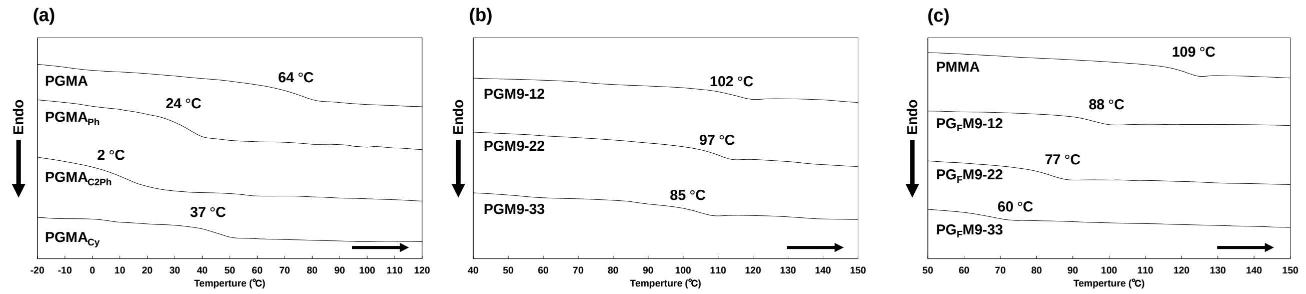

Supplementary Figure 19 | DSC curves of the (a) PGMA and its derivatives, (b) PGMs, and (c) PMMA (M_n = 8.60 kg mol^{-1}, $Đ$ = 1.03) prepared through living anionic polymerization and PG$_F$Ms in the second heating cycle.

Supplementary Table 5 | 5% weight loss temperature and glass transition temperature of the synthesized PGMA, PGMs, and their derivatives

Sample	5% weight loss temperature[a] (°C)	T_g[b] (°C)
PGMA	328	64
PGMA$_{Ph}$	301	24
PGMA$_{C2Ph}$	297	2
PGMA$_{Cy}$	296	37
PGM9-12	326	102
PGM9-22	326	97
PGM9-33	319	85
PG$_F$M9-12	330	88
PG$_F$M9-22	328	77
PG$_F$M9-33	320	60

[a]Determined by thermal gravity analysis.

[b]Determined from baseline shift of second heating cycle in DSC measurement.

Supplementary Table 6 | Contact angles (C.A.) and surface free energies (SFEs) of PG$_F$Ms

Sample	PGMA$_F$ (mol%)	C.A. (H_2O)[a] (°)	C.A. (CH_2I_2)[a] (°)	SFE[b] (mJ m^{-2})
PG$_F$M9-33	33	77.0	51.8	36.5
PG$_F$M9-22	22	77.9	48.7	37.5
PG$_F$M9-12	12	74.5	45.3	40.0
PS	–	–	–	40.7[2]

[a]Average values of measurements taken at 6 positions.

[b]SFEs of PG$_F$Ms were calculated by Owens–Wendt method.

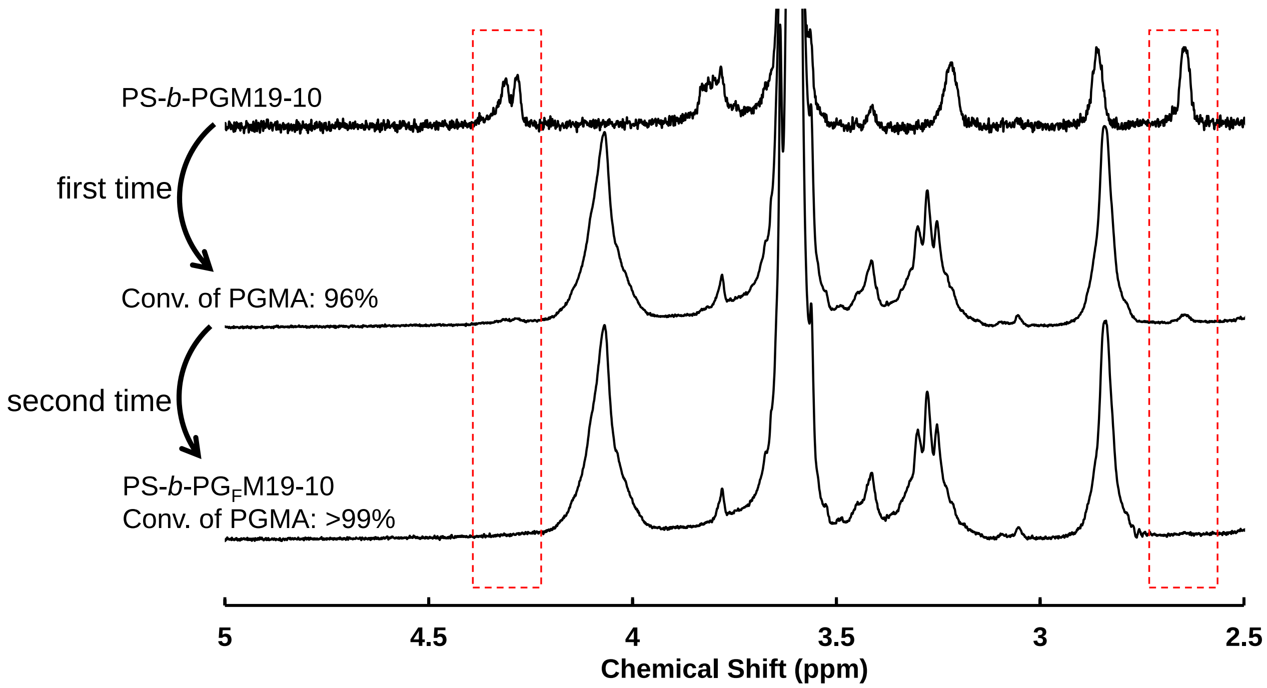

Supplementary Figure 20 | The ^{1}H NMR spectra ($CDCl_3$) of PS-*b*-PGM19-10 (top), a PS-*b*-PGM19-10 derivative obtained after the first thiol–epoxy reaction (middle), and the objective PS-*b*-PG$_F$M19-10 (bottom) for monitoring the conversion of PGMA.

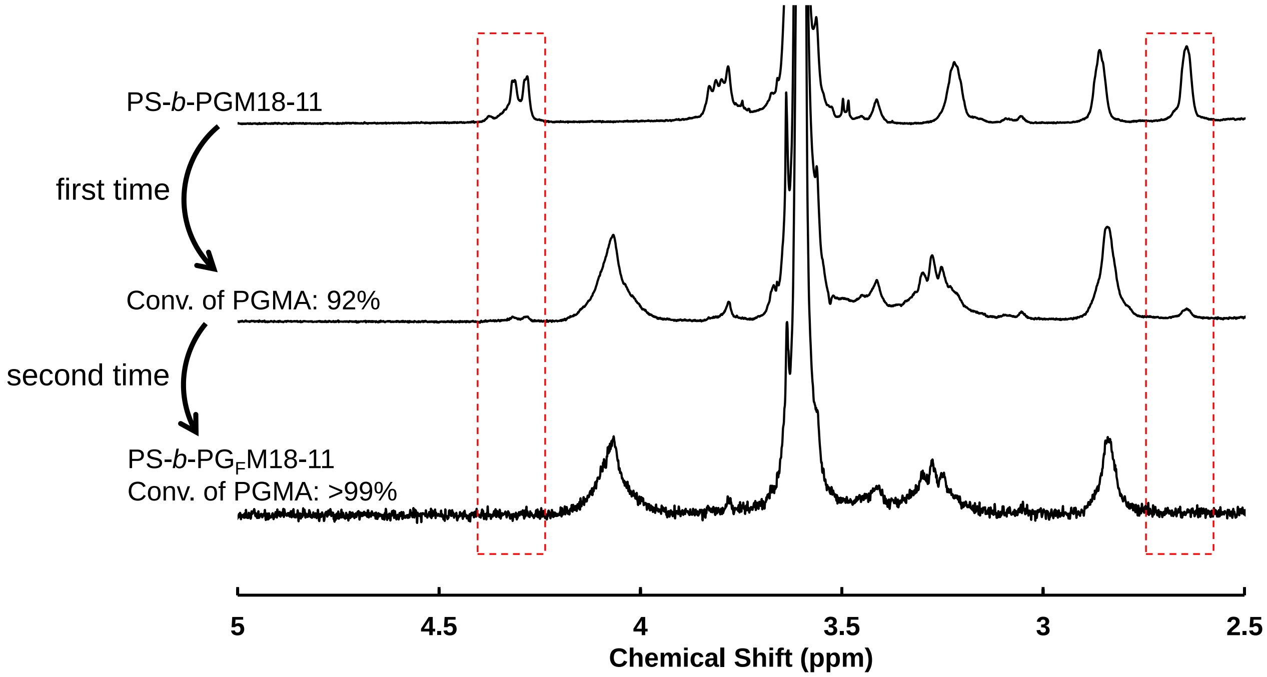

Supplementary Figure 21 | The ^{1}H NMR spectra ($CDCl_3$) of PS-*b*-PGM18-11 (top), a PS-*b*-PGM18-11 derivative obtained after the first thiol–epoxy reaction (middle), and the objective PS-*b*-PG$_F$M18-11 (bottom) for monitoring the conversion of PGMA.

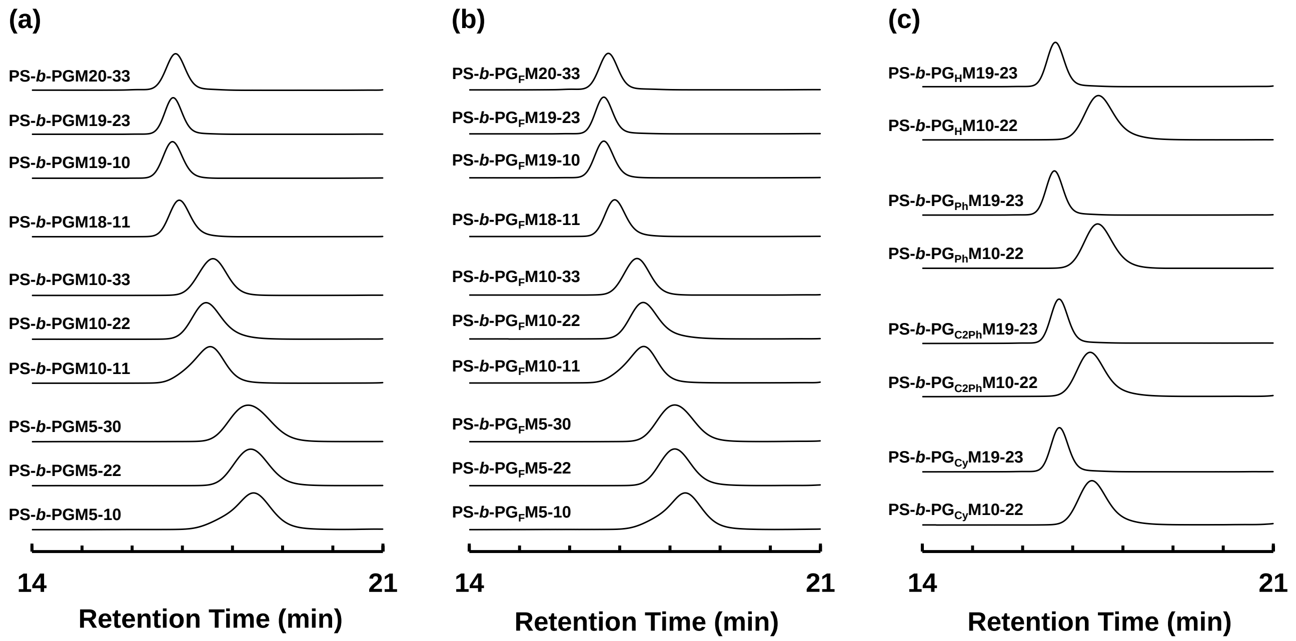

Supplementary Figure 22 | SEC traces of the (a) PS-*b*-PGMs, (b) PS-*b*-PG$_F$Ms, and (c) PS-*b*-PG$_R$Ms in THF.

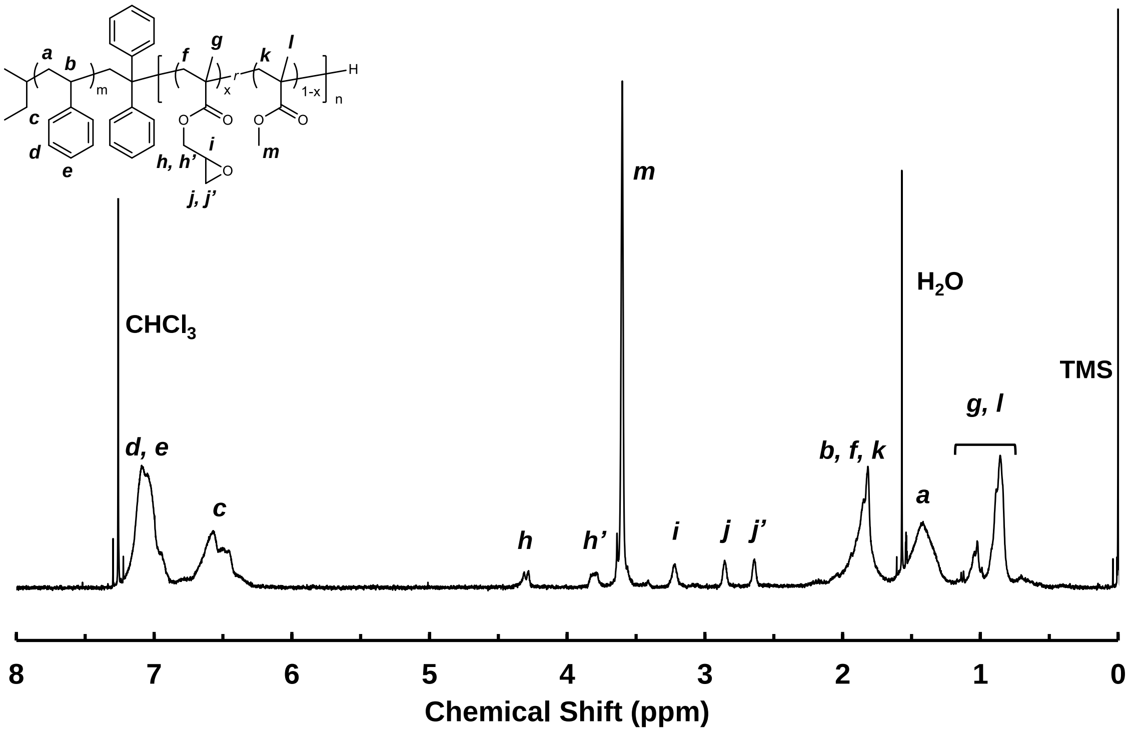

Supplementary Figure 23 | ^{1}H NMR spectrum of PS-*b*-PGM10-22 in $CDCl_3$.

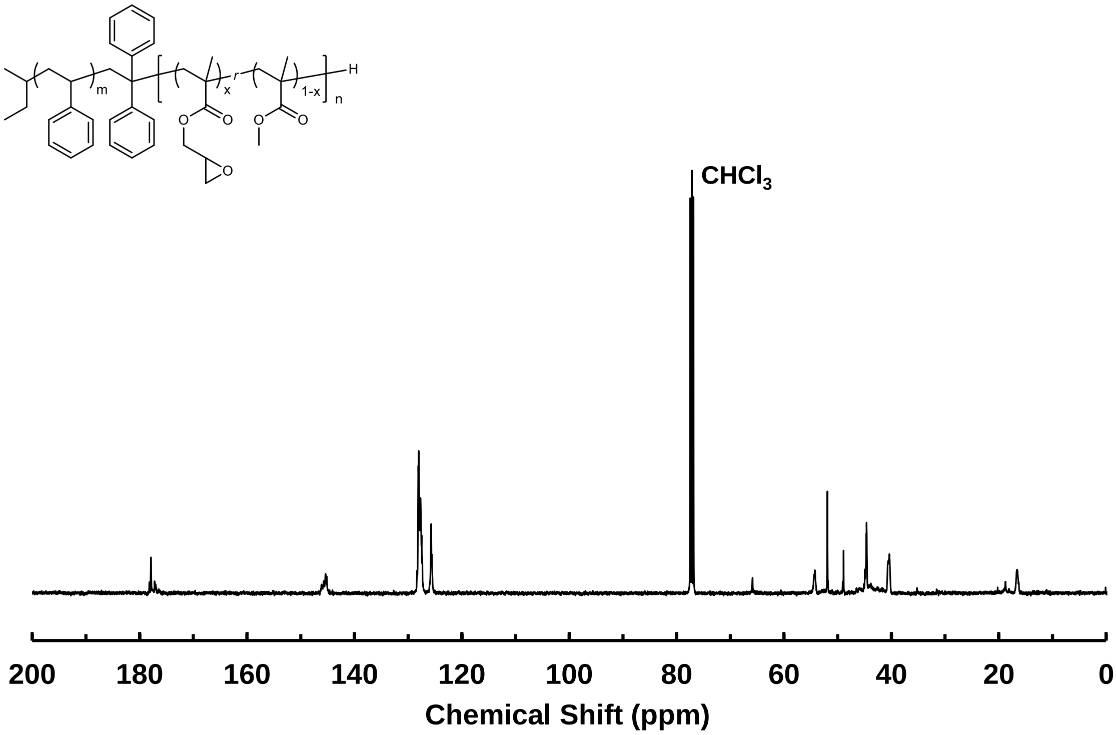

Supplementary Figure 24 | ^{13}C NMR spectrum of PS-*b*-PGM10-22 in $CDCl_3$.

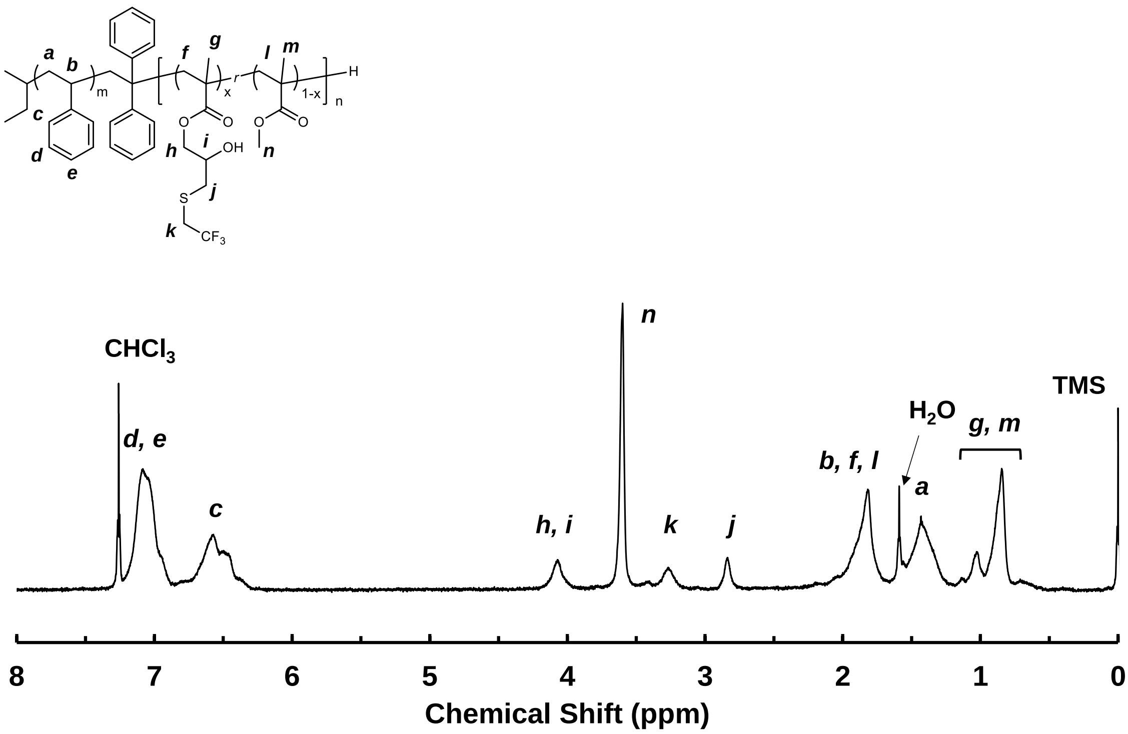

Supplementary Figure 25 | ^{1}H NMR spectrum of PS-*b*-PG$_F$M10-22 in $CDCl_3$.

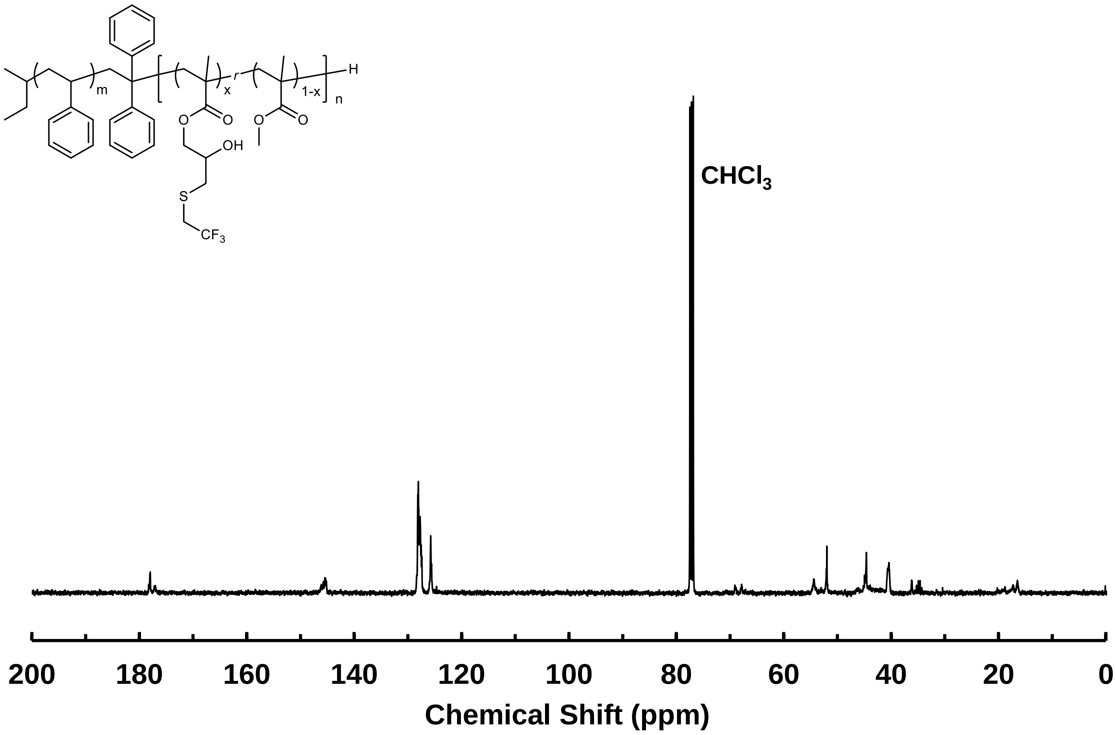

Supplementary Figure 26 | ^{13}C NMR spectrum of PS-*b*-PG$_F$M10-22 in $CDCl_3$.

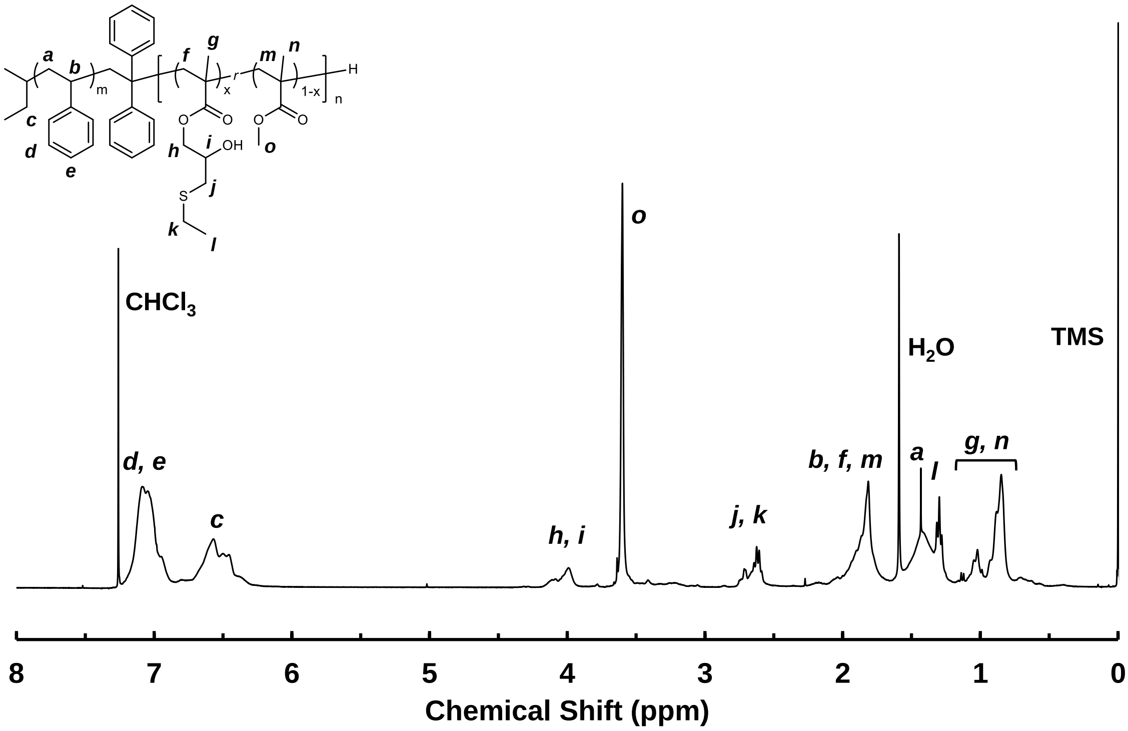

Supplementary Figure 27 | ^{1}H NMR spectrum of PS-*b*-PG$_{H}$M10-22 in $CDCl_3$.

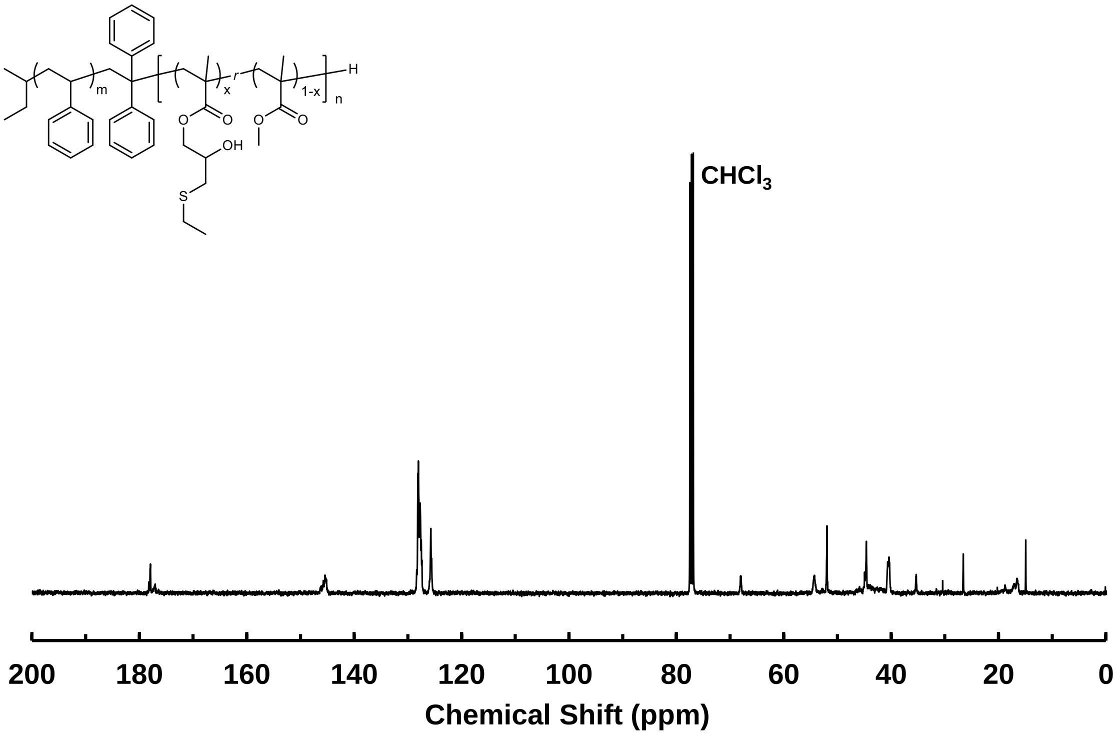

Supplementary Figure 28 | ^{13}C NMR spectrum of PS-*b*-PG$_{H}$M10-22 in $CDCl_3$.

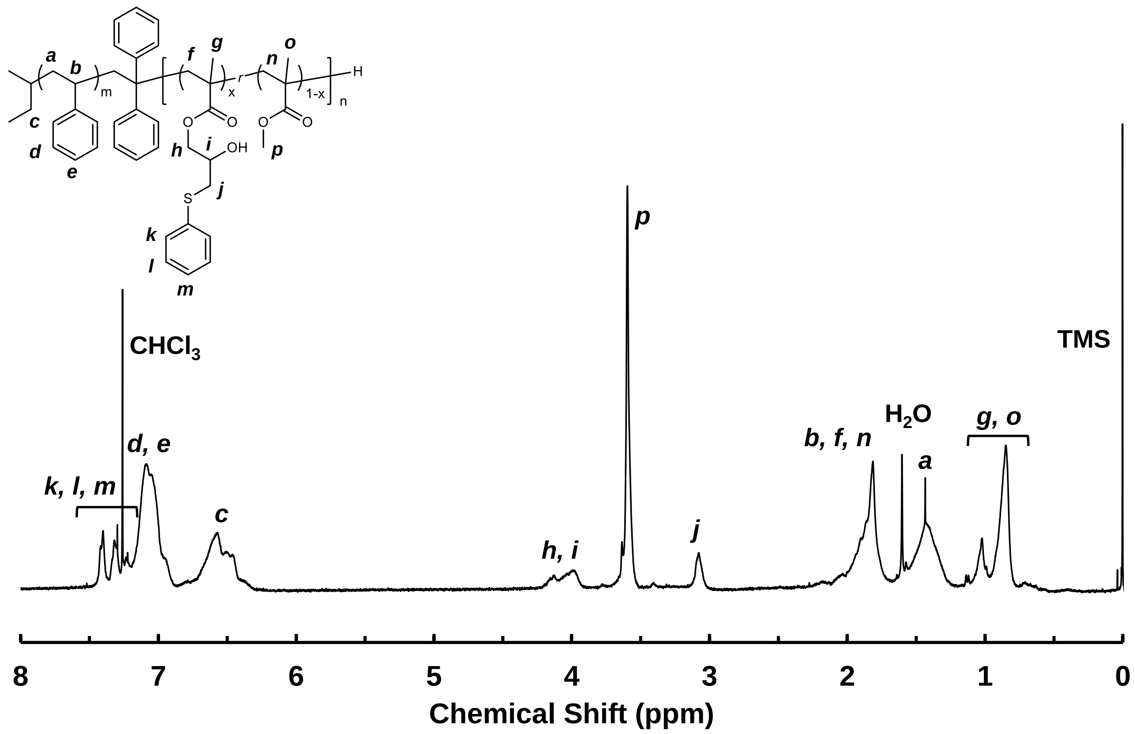

Supplementary Figure 29 | ^{1}H NMR spectrum of PS-*b*-PG$_{Ph}$M10-22 in $CDCl_3$.

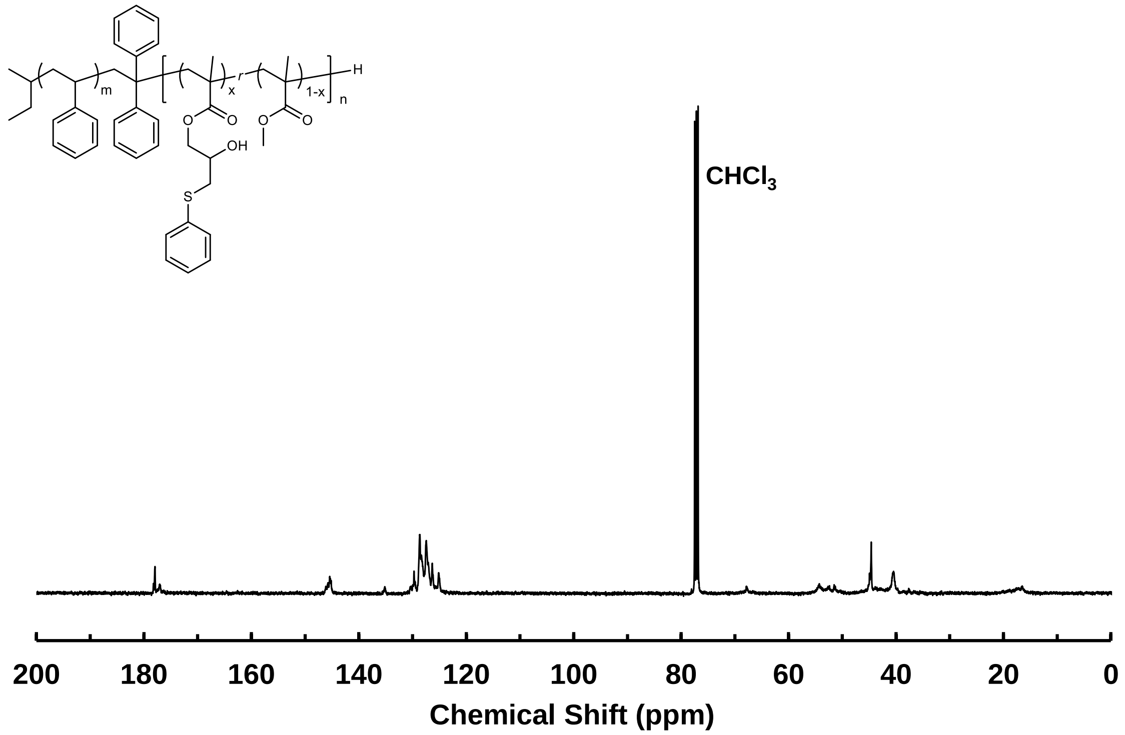

Supplementary Figure 30 | ^{13}C NMR spectrum of PS-*b*-PG$_{Ph}$M10-22 in $CDCl_3$.

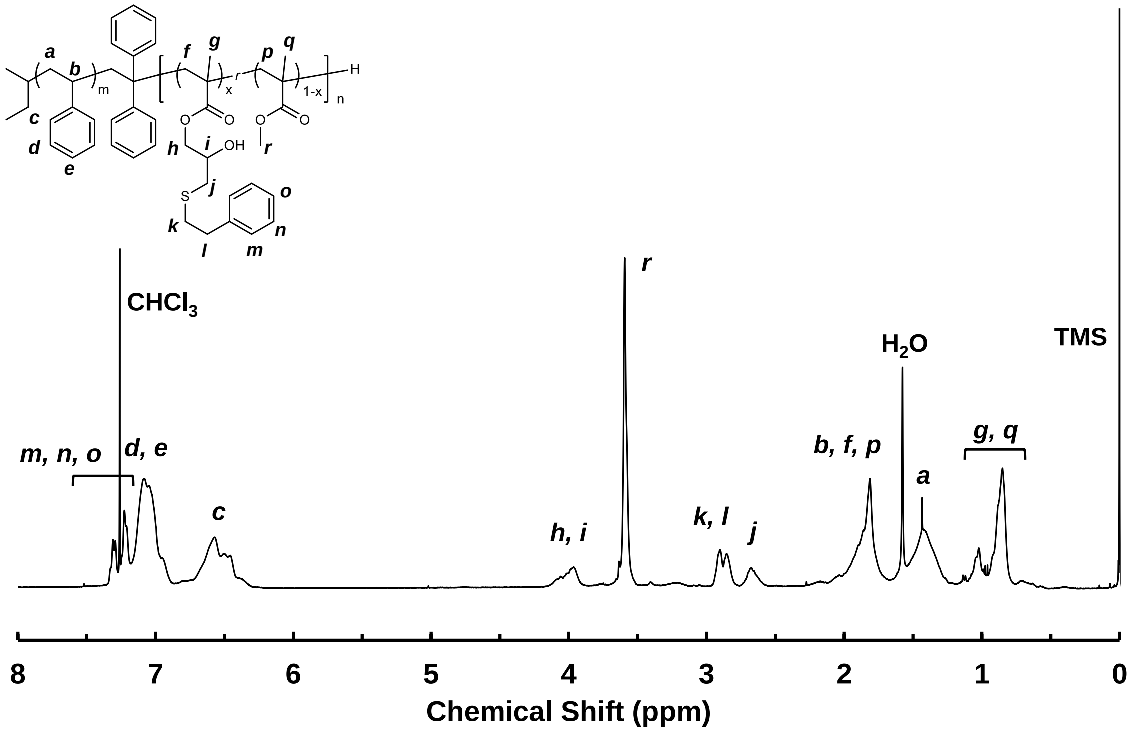

Supplementary Figure 31 | ^{1}H NMR spectrum of PS-*b*-PG$_{C2Ph}$M10-22 in $CDCl_3$.

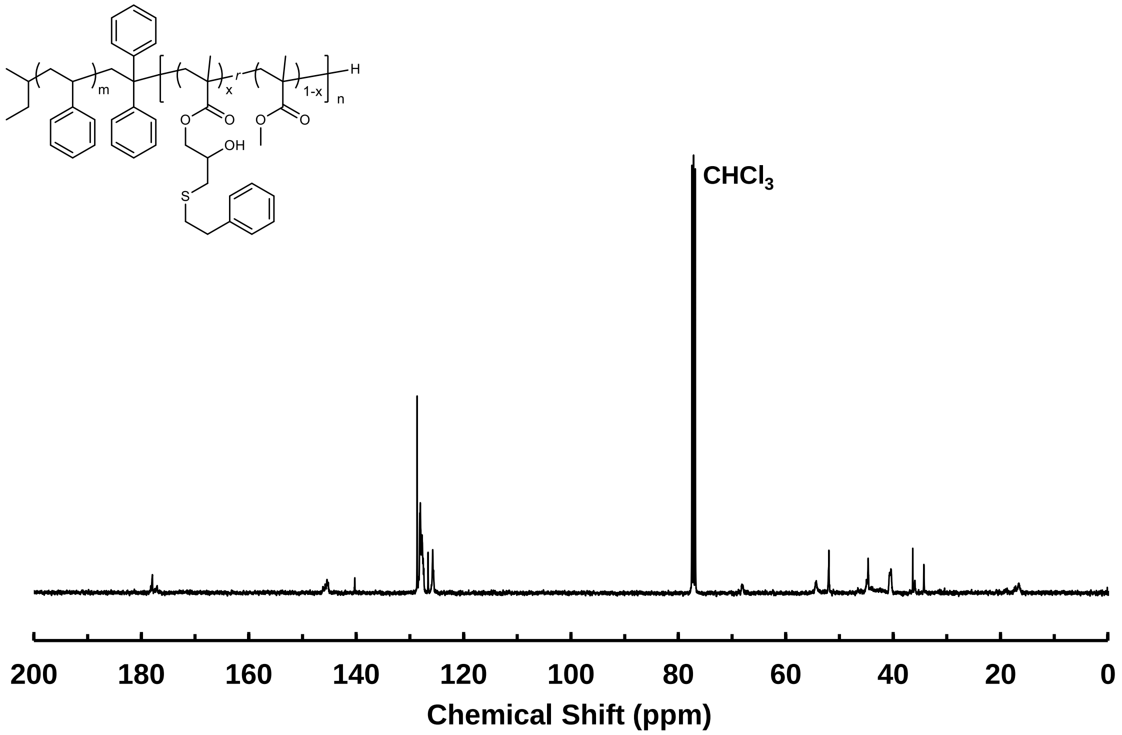

Supplementary Figure 32 | ^{13}C NMR spectrum of PS-*b*-PG$_{C2Ph}$M10-22 in $CDCl_3$.

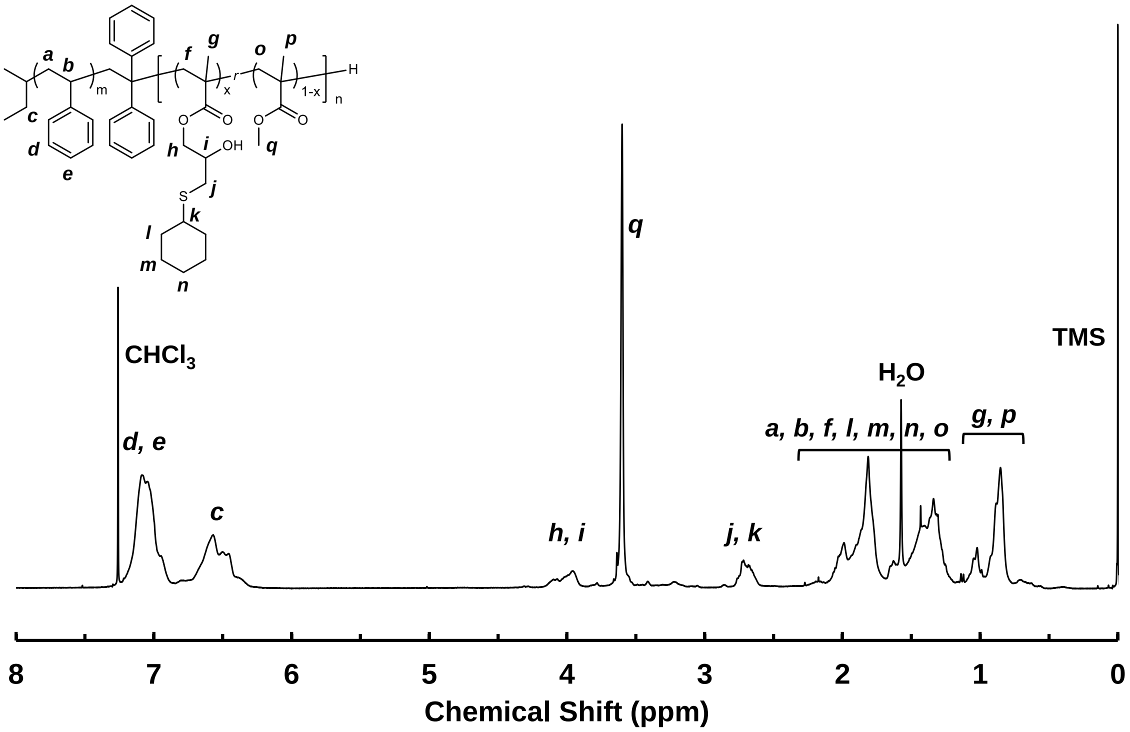

Supplementary Figure 33 | ^{1}H NMR spectrum of PS-*b*-PG$_{Cy}$M10-22 in $CDCl_3$.

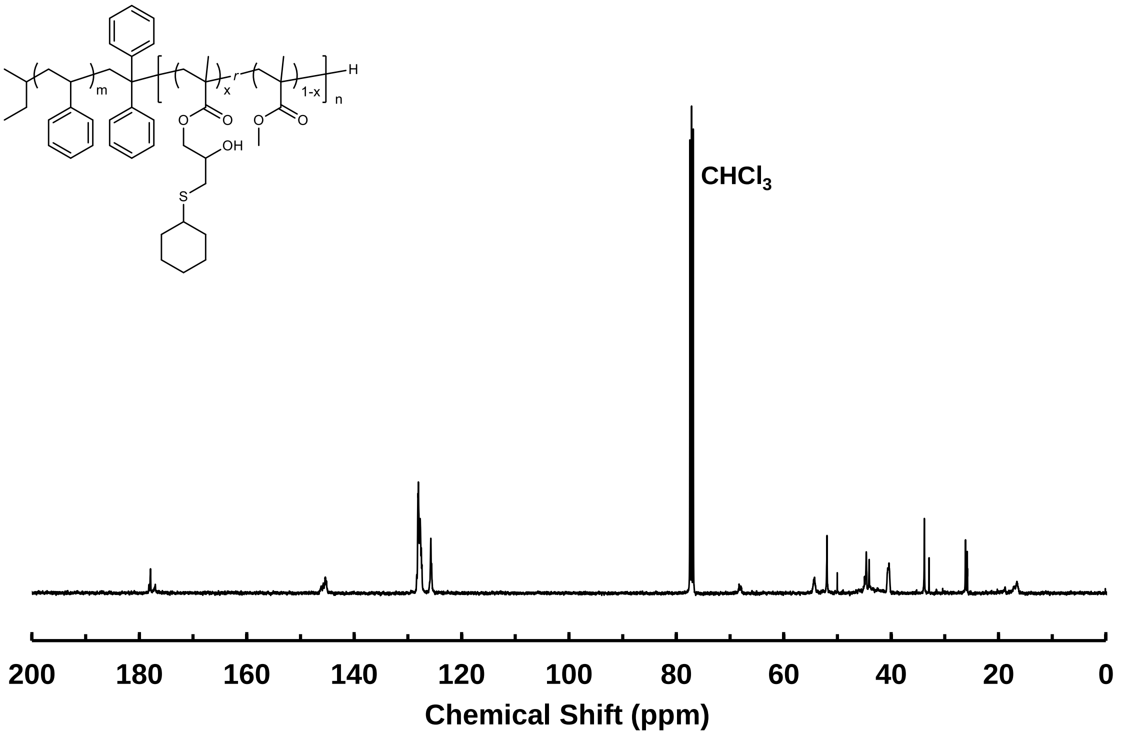

Supplementary Figure 34 | ^{13}C NMR spectrum of PS-*b*-PG$_{Cy}$M10-22 in $CDCl_3$.

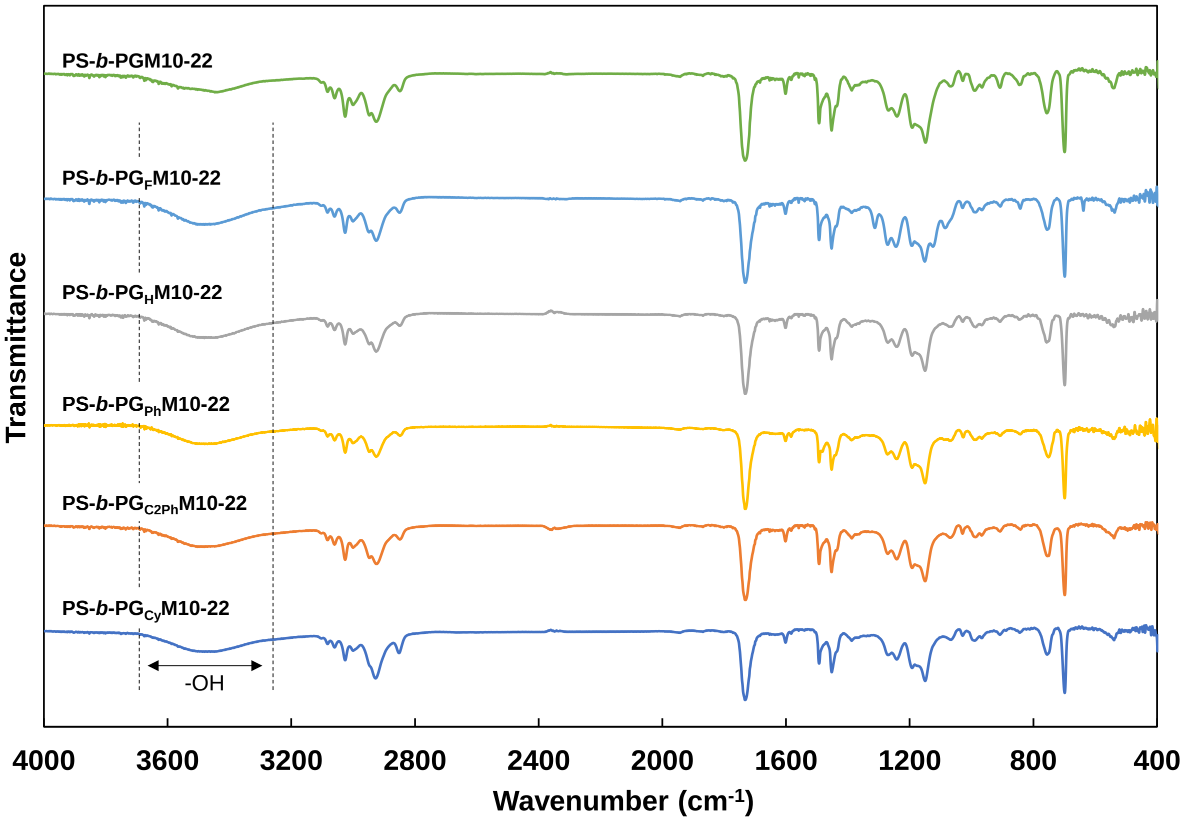

Supplementary Figure 35 | FT-IR spectra of the synthesized BCPs.

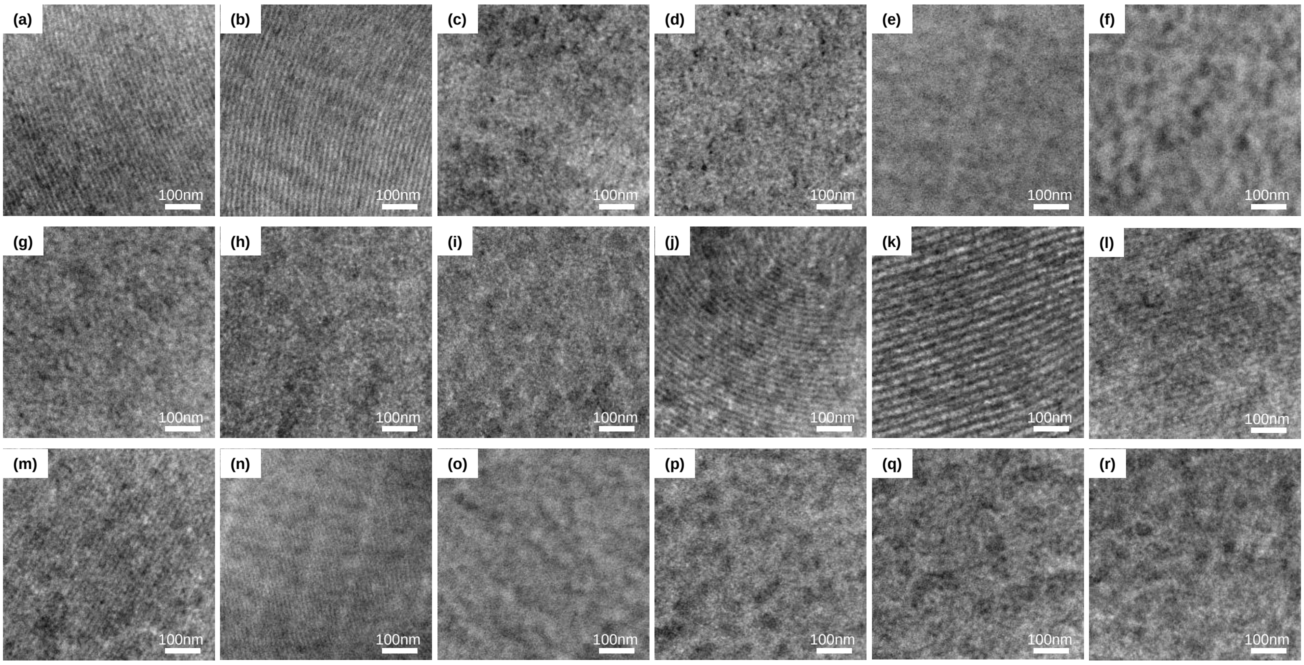

Supplementary Figure 36 | TEM images of (a) PS-*b*-PGM20-33, (b) PS-*b*-PGM19-23, (c) PS-*b*-PGM19-10, (d) PS-*b*-PGM18-11, (e) PS-*b*-PGM10-33, (f) PS-*b*-PGM10-11, (g) PS-*b*-PGM5-30, (h) PS-*b*-PGM5-22, (i) PS-*b*-PGM5-10, (j) PS-*b*-PG$_{F}$M20-33, (k) PS-*b*-PG$_{F}$M19-23, (l) PS-*b*-PG$_{F}$M19-10, (m) PS-*b*-PG$_{F}$M18-11, (n) PS-*b*-PG$_{F}$M10-33, (o) PS-*b*-PG$_{F}$M10-11, (p) PS-*b*-PG$_{F}$M5-30, (q) PS-*b*-PG$_{F}$M5-22, and (r) PS-*b*-PG$_{F}$M5-10. The dark regions correspond to the PS block stained with RuO_4.

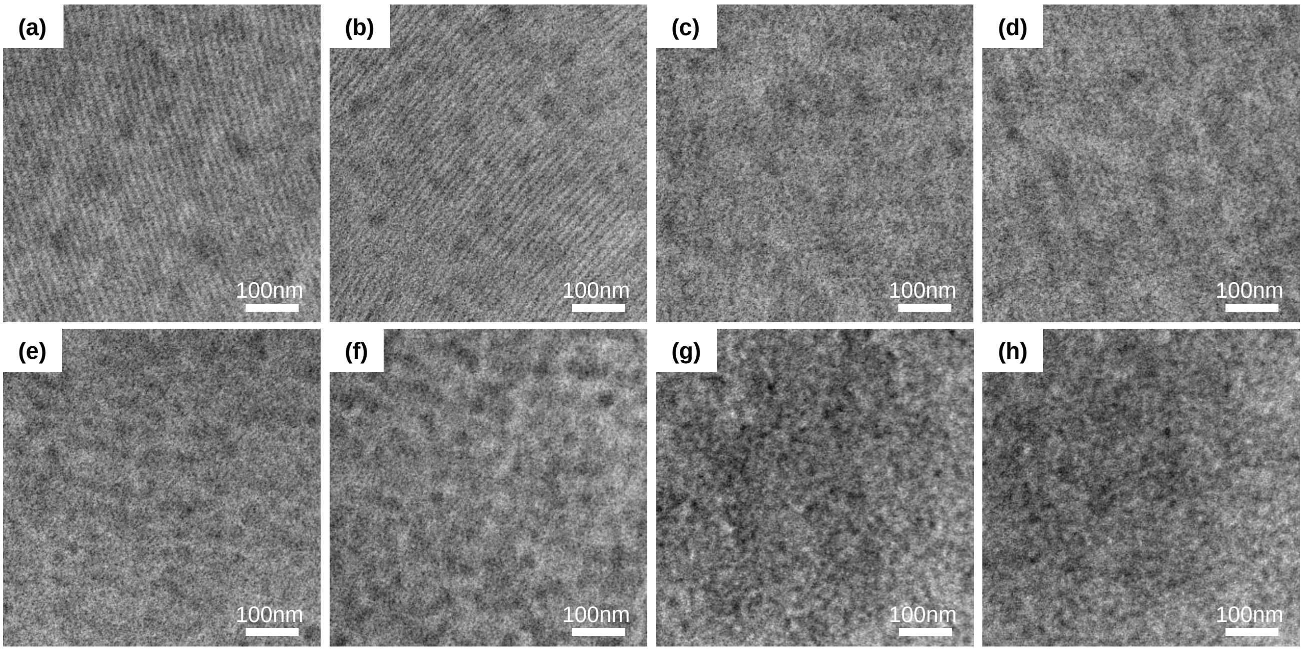

Supplementary Figure 37 | TEM images of (a) PS-*b*-PG$_{H}$M19-23, (b) PS-*b*-PG$_{Ph}$M19-23, (c) PS-*b*-PG$_{C2Ph}$M19-23, (d) PS-*b*-PG$_{Cy}$M19-23, (e) PS-*b*-PG$_{H}$M10-22, (f) PS-*b*-PG$_{Ph}$M10-22, (g) PS-*b*-PG$_{C2Ph}$M10-22, and (h) PS-*b*-PG$_{Cy}$M10-22. The dark regions correspond to the PS block stained with RuO_4.

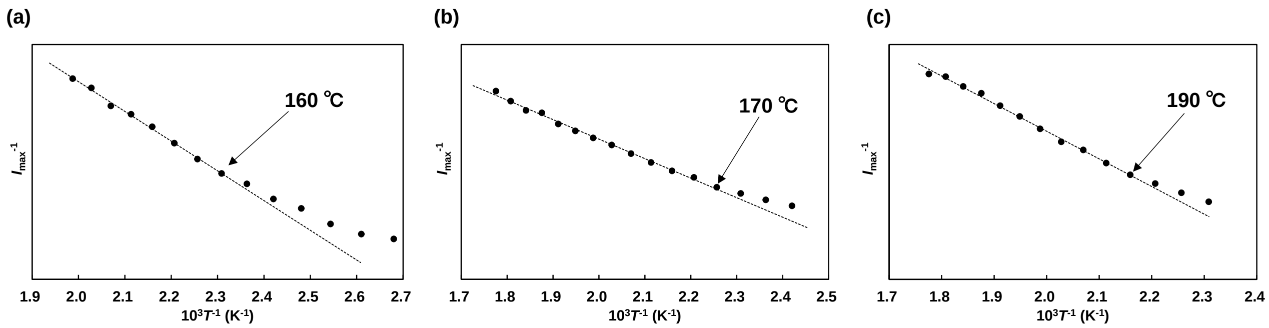

Supplementary Figure 38 | The I_{max}^{-1}–T^{-1} plots for (a) PS-*b*-PG$_F$M5-10, (b) PS-*b*-PG$_F$M5-22, and (c) PS-*b*-PG$_F$M5-30. I_{max} is the maximum value of intensity in the SAXS profile at each temperature. The temperatures noted are attributed to mean-field to non-mean-field transitions of disordered states at 150–160, 160–170, and 180–190 °C for (a) PS-*b*-PG$_F$M5-10, (b) PS-*b*-PG$_F$M5-22, and (c) PS-*b*-PG$_F$M5-30, respectively.

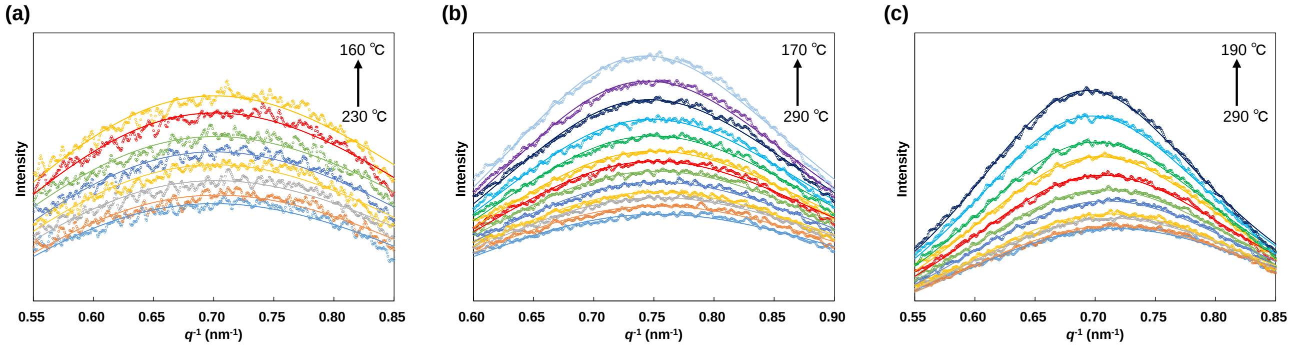

Supplementary Figure 39 | The scattering profiles measured by SAXS (dots) and the fitting profiles (lines) at different temperatures for (a) PS-*b*-PG$_F$M5-10, (b) PS-*b*-PG$_F$M5-22, and (c) PS-*b*-PG$_F$M5-30.

Supplementary Table 7 | The fixed parameters used to determine χ_{eff} for each PS-*b*-PG$_F$M

Sample	r_c	M_n[a] (g mol^{-1})	M_w (g mol^{-1})	$M_{n, PS}$ (g mol^{-1})	f_{PS}	w_{PS}	ρ_{PGFM}[b] (g cm^{-3})	FW_{PGFM}[c] (g mol^{-1})
PS-*b*-PG$_F$M5-30	71	5782	6302	2978	0.570	0.515	1.255	147.6
PS-*b*-PG$_F$M5-22	72	5822	6288	2946	0.555	0.506	1.235	134.9
PS-*b*-PG$_F$M5-10	64	5152	5822	2241	0.475	0.435	1.205	115.9

[a]The molecular weights of *sec*-Butyl group and DPE were excluded.

[b]The densities of PG$_F$Ms were calculated from the composition ratios of PG$_F$Ms based on the densities of 1.18 g cm^{-3} for PMMA and 1.43 g cm^{-3} for PGMA$_F$.

[c]The formula weights of PG$_F$M were calculated from the composition ratios of PG$_F$Ms based on the formula weights of 100.12 g mol^{-1} for MMA and 258.26 g mol^{-1} for GMA$_F$.

Supplementary Table 8 | The determined χ_{eff} values for each sample at different temperatures

T (°C)	χ_{eff} of PS-*b*-PG$_F$M5-10[a] (RPA)	χ_{eff} of PS-*b*-PG$_F$M5-10[b] (calculation)	χ_{eff} of PS-*b*-PG$_F$M5-22[a] (RPA)	χ_{eff} of PS-*b*-PG$_F$M5-22[b] (calculation)	χ_{eff} of PS-*b*-PG$_F$M5-30[a] (RPA)	χ_{eff} of PS-*b*-PG$_F$M5-30[b] (calculation)
160	0.114	0.114	–	–	–	–
170	0.113	0.113	0.136	0.136	–	–
180	0.110	0.112	0.135	0.135	–	–
190	0.111	0.111	0.134	0.134	0.142	0.143
200	0.111	0.110	0.134	0.133	0.142	0.142
210	0.109	0.109	0.133	0.133	0.141	0.141
220	0.109	0.108	0.132	0.132	0.141	0.140
230	0.107	0.107	0.131	0.131	0.140	0.140
240	–	–	0.130	0.130	0.139	0.139
250	–	–	0.130	0.129	0.139	0.138
260	–	–	0.129	0.129	0.138	0.138
270	–	–	0.128	0.128	0.137	0.137
280	–	–	0.127	0.127	0.137	0.137
290	–	–	0.126	0.126	0.136	0.136

[a]Determined by the RPA method.

[b]Calculated by $\chi_{eff} = \alpha + \beta/T$.

Supplementary Table 9 | Determined entropic and enthalpic contributions for PS-*b*-PMMA, PS-*b*-PG$_F$Ms, and PS-*b*-PHFMA

Sample	α	β
PS-*b*-PMMA[3]	0.024	3.21
PS-*b*-PG$_F$M5-10	0.0691	19.3
PS-*b*-PG$_F$M5-22	0.0893	20.9
PS-*b*-PG$_F$M5-30	0.105	17.4
PS-*b*-PHFMA[1]	0.126	19.4

Supplementary Table 10 | χN values of PS-*b*-PG$_F$Ms

Sample	χN[a]	Higher-order structure in bulk[b]
PS-*b*-PG$_F$M20-33	35.8	Ordered
PS-*b*-PG$_F$M19-23	33.0	Ordered
PS-*b*-PG$_F$M19-10	26.8	Ordered
PS-*b*-PG$_F$M18-11	23.3	Ordered
PS-*b*-PG$_F$M10-33	17.2	Ordered
PS-*b*-PG$_F$M10-22	15.8	Ordered
PS-*b*-PG$_F$M10-11	13.5	Disordered
PS-*b*-PG$_F$M5-30	8.6	Disordered
PS-*b*-PG$_F$M5-22	7.9	Disordered
PS-*b*-PG$_F$M5-10	6.4	Disordered

[a]The χ values for each of PS-*b*-PG$_F$M with 10, 20, and 30mol% PGMA was 0.110, 0.133, and 0.142, respectively. N was calculated in the same way as r_c. [b]Determined by SAXS and TEM.

In this study, the instrumental broadening factor was not considered. However, the χ_{eff} values of PS-*b*-PMMA estimated using the same SAXS instrument were from 0.031 to 0.037 at 200 °C [3] and these values were in good agreement with the χ_{eff} values of earlier studies that were from 0.028 to 0.054 at 200 °C[4–8]. Supplementary Table 10 shows estimated χN values and observed higher-order structures in bulk of PS-*b*-PG$_F$Ms. These results suggest that the critical value of χN for order–disorder transition (χN_{ODT}) of PS-*b*-PG$_F$M is around 10.5 and observed structures do not contradict the expected structure except for PS-*b*-PG$_F$M10-11. The considered reasons why a disordered structure was formed despite the χN of PS-*b*-PG$_F$M10-11 being larger than 10.5 are that the volume fraction of PS in the polymer was not exactly 0.5, and the χN_{ODT} value of PS-*b*-PG$_F$M is higher than the theoretical value.

Supplementary Table 11 | Characterization data for synthesized PS-*r*-PMMA-*r*-PHEMA random copolymers

Sample	Reaction time (h)	M_n[a] (kg mol^{-1})	$Đ$[a]	PS molar ratio[b]	PMMA molar ratio[b]	PHEMA molar ratio
NL35	8	22.9	1.85	0.35	0.60	0.05
NL38	8	23.6	1.75	0.38	0.57	0.05
NL46	24	30.6	2.58	0.46	0.49	0.05
NL50	24	27.9	2.42	0.50	0.45	0.05

[a]Determined by SEC in THF against PS standards.

[b]Determined by ^{1}H NMR spectroscopy in $CDCl_3$.

Supplementary Table 12 | Conditions of the prepared thin films for the samples shown in Figure 5

Sample	Random copolymer	Film thickness of BCP (nm)
PS-*b*-PG$_F$M20-33	NL46	25.3
PS-*b*-PG$_F$M19-23	NL38	25.6
PS-*b*-PG$_F$M19-10	NL46	25.9
PS-*b*-PG$_F$M18-11	NL50	19.5
PS-*b*-PG$_F$M10-33	NL46	17.6
PS-*b*-PG$_F$M10-22	NL50	16.1

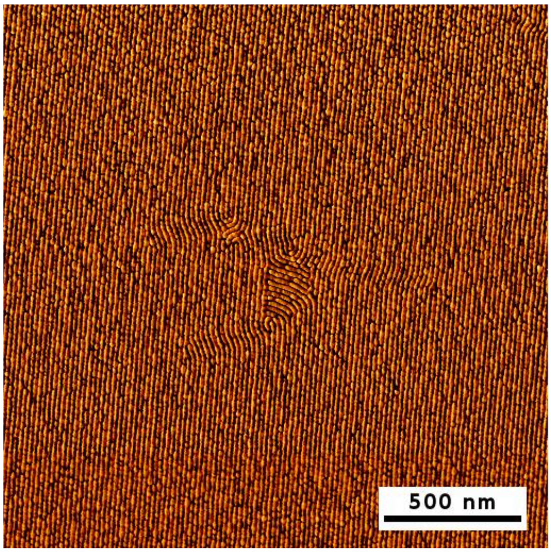

Supplementary Figure 40 | An AFM phase image of a PS-*b*-PG$_F$M19-23 film on an NL-35-modified DSA substrate (L_s = 90 nm) after annealing at 240 °C for 5 min. This image shows structural defects of the line pattern in PS-*b*-PG$_F$M19-23 thin films.

Supplementary References

1. Yoshimura, Y., Chandra, A., Nabae, Y. & Hayakawa, T. Chemically tailored high-χ block copolymers for perpendicular lamellae *via* thermal annealing. *Soft Matter* **15**, 3497–3506 (2019). 10.1039/c9sm00128j, Pubmed:30855615.
2. Wu, S. Surface and interfacial tensions of polymer melts. II. Poly(methyl methacrylate), poly(*n*-butyl methacrylate), and polystyrene. *J. Phys. Chem.* **74**, 632–638 (1970). 10.1021/j100698a026.
3. Agata, Y., Nabae, Y., Ougizawa, T. & Hayakawa, T. Effect of modified chemical junction of a diblock copolymer on the microphase separation behavior. *J. Photopolym. Sci. Technol.* **35**, 17–22 (2022). 10.2494/photopolymer.35.17.
4. Russell, T. P., Hjelm, R. P. & Seeger, P. A. Temperature dependence of the interaction parameter of polystyrene and poly(methyl methacrylate). *Macromolecules* **23**, 890–893 (1990). 10.1021/ma00205a033.
5. Stühn, B. The relation between the microphase separation transition and the glass transition in diblock copolymers. *J. Polym. Sci. Part B Polym. Phys.* **30**, 1013–1019 (1992). 10.1002/polb. 1992.090300909.
6. Callaghan, T. A. & Paul, D. R. Interaction energies for blends of poly(methyl methacrylate), polystyrene, and poly(α-methylstyrene) by the critical molecular weight method. *Macromolecules* **26**, 2439–2450 (1993). 10.1021/ma00062a008.
7. Russell, T. P. Changes in polystyrene and poly(methyl methacrylate) interactions with isotopic substitution. *Macromolecules* **26**, 5819–5819 (1993). 10.1021/ma00073a044.
8. Zhao, Y., Sivaniah, E. & Hashimoto, T. SAXS analysis of the order−disorder transition and the interaction parameter of polystyrene-*block*-poly(methyl methacrylate). *Macromolecules* **41**, 9948–9951 (2008). 10.1021/ma8013004.
